# Expanded roles of leucine-responsive regulatory protein in transcription regulation of the *Escherichia coli* genome: Genomic SELEX screening of the regulation targets

**DOI:** 10.1099/mgen.0.000001

**Published:** 2015-07-15

**Authors:** Tomohiro Shimada, Natsumi Saito, Michihisa Maeda, Kan Tanaka, Akira Ishihama

**Affiliations:** 1 Chemical Resources Laboratory, Tokyo Institute of Technology, Nagatsuta, Yokohama, Japan; 2 Department of Frontier Bioscience, Hosei University, Koganei, Tokyo, Japan; 3 Research Center for Micro-Nano Technology, Hosei University, Koganei, Tokyo, Japan; 4 Department of Chemistry and Material Engineering, Tsuruoka National College of Technology, Yamagata, Japan; 5 Institute for Advanced Biosciences, Keio University, Yamagata, Japan; 6 School of Agriculture, Meiji University, Kawasaki, Kanagawa, Japan

**Keywords:** *Escherichia coli* genome, Genomic SELEX, leucine response regulator, regulation target, transcription factor

## Abstract

Leucine-responsive regulatory protein (Lrp) is a transcriptional regulator for the genes involved in transport, biosynthesis and catabolism of amino acids in *Escherichia coli*. In order to identify the whole set of genes under the direct control of Lrp, we performed Genomic SELEX screening and identified a total of 314 Lrp-binding sites on the *E. coli* genome. As a result, the regulation target of Lrp was predicted to expand from the hitherto identified genes for amino acid metabolism to a set of novel target genes for utilization of amino acids for protein synthesis, including tRNAs, aminoacyl-tRNA synthases and rRNAs. Northern blot analysis indicated alteration of mRNA levels for at least some novel targets, including the aminoacyl-tRNA synthetase genes. Phenotype MicroArray of the *lrp* mutant indicated significant alteration in utilization of amino acids and peptides, whilst metabolome analysis showed variations in the concentration of amino acids in the *lrp* mutant. From these two datasets we realized a reverse correlation between amino acid levels and cell growth rate: fast-growing cells contain low-level amino acids, whilst a high level of amino acids exists in slow-growing cells. Taken together, we propose that Lrp is a global regulator of transcription of a large number of the genes involved in not only amino acid transport and metabolism, but also amino acid utilization.

## Impact Statement

Leucine-responsive regulatory protein (Lrp) is known as a global regulator of the genes for transport, biosynthesis and catabolism of amino acids to establish their balance needed for protein synthesis. After Genomic SELEX screening, however, we identified that Lrp not only controls the production of amino acids, but also the utilization pathway of amino acids by regulating the genes for tRNAs, aminoacyl-tRNA synthetases and rRNAs. Phenotype MicroArray and metabolome analyses indicated Lrp-mediated correlation between the intracellular levels of amino acids and their utilization for protein synthesis: the intracellular levels are low for amino acids that are efficiently used for protein synthesis, allowing fast cell growth, but cell growth is low even in the presence of high levels of amino acids that are not so much used for protein synthesis. Here, we also identified another expanded role of Lrp in regulation of a set of transcription factors, each playing a regulatory role in the control of a specific metabolism pathway or physiological response to a specific nutritional condition. Lrp stays on the top of this hierarchic network of transcription factors. Overall, we propose an expanded role for Lrp in controlling the production and utilization of amino acids – the key metabolites of cell construction.

## Introduction

Leucine-responsive regulatory protein (Lrp) belongs to the widely distributed Lrp–AsnC family of small, basic transcription factors. *Escherichia coli* Lrp of 164 aa in size consists of three functional domains: an N-terminal 40  % domain containing the helix–turn–helix motif of DNA binding, the next 40  % of the middle domain responsible for transcription activation and an overlapping C-terminal domain required for the response to Leu (de los Rios & Perona, 2007; Ettema *et al.*, 2002; Platko & Calvo, 1993). Lrp forms a dimer in solution (Calvo & Matthews, 1994; Willins *et al.*, 1991), but self-assembles to form a mixture of octamers and hexadecamers (Chen *et al.*, 2001b). As Lrp-regulated promoters commonly contain multiple adjacent Lrp-binding sites, the higher-order structures could play an important functional role.

Lrp was first identified in *E. coli* as a regulatory protein involved in the control of the transport of branched-chain amino acids (Anderson *et al.*, 1976). Subsequently, mutations in the *lrp* gene were found to influence the expression of operons involved in the biosynthesis and degradation of some more amino acids (Lin *et al.*, 1992; Platko *et al.*, 1993), suggesting that Lrp plays a regulatory role in transport and metabolism of not only Leu, but also some other amino acids. The number of regulation targets of Lrp has further increased concomitant with the advance of genome expression monitoring systems. Proteome analysis suggested the alteration of levels of a total of 25 proteins in the *lrp* mutant (Ernsting *et al.*, 1992). The alteration of expression levels of up to 85 proteins was also identified by random phage insertions into the genome (Lin *et al.*, 1992). The transcriptome analysis indicated that as many as >400 genes or ∼10  % of the genes within the *E. coli* genome are affected in the absence of Lrp, of which at least 130 were suggested to be under the direct control of Lrp (Cho *et al.*, 2008; Hung *et al.*, 2002; Tani *et al.*, 2002). A certain proportion of the regulated genes are involved, as originally proposed, in transport and metabolism of amino acids, but Lrp has also been suggested to regulate genes involved in biosynthesis and degradation of various metabolites other than amino acids (Brinkman *et al.*, 2003; Calvo & Matthews, 1994; Newman & Lin, 1995). In addition, the genes for other cellular functions, such as pili synthesis and adhesion to host cells, have been indicated to be under the control of Lrp (Calvo & Matthews, 1994). Furthermore, Lrp is also known to function as a structural element, together with other the nucleoid proteins, to establish the conformation of genome DNA (reviewed by Ishihama, 2009). Thus, as in the case of other nucleoid proteins, Lrp is a bifunctional protein, playing a regulatory role in gene expression and an architectural role in nucleoid organization. Accordingly, the intracellular level of Lrp in exponentially growing *E. coli* cells is as abundant as other nucleoid proteins (Ali Azam *et al.*, 1999; Ishihama *et al.*, 2014; Willins *et al.*, 1991).

One unique characteristic of Lrp is its functional modulation after interaction with multiple effectors. The regulatory function of Lrp was first recognized under the control of Leu (Chen & Calvo, 2002; Chen *et al.*, 2001a; Haney *et al.*, 1992; Platko & Calvo, 1993; Roesch & Blomfield, 1998; Willins *et al.*, 1991). Leu is the most abundant building block (∼9  % of total blocks) of all proteins in *E. coli*, suitable as a representative signal molecule of the availability of substrates for protein production. Lrp acts as a sensor of this key signal, leading to modulation of its activity and specificity. The effector Leu modulates multimerization of Lrp and thereby controls the transcription of certain target genes (Chen & Calvo, 2002; Chen *et al.*, 2001a, b). In most cases, Lrp has been reported to activate the operons that encode enzymes for amino acid biosynthesis and repress the operons that encode catabolic enzymes (Calvo & Matthews, 1994). The activation of some operons is overcome by Leu, but in other cases the activation requires Leu (Calvo & Matthews, 1994; Ernsting *et al.*, 1992; Lin *et al.*, 1992; Newman *et al.*, 1992). A group of regulation target genes are, however, activated by Lrp independent of Leu. More complexity has arisen from the findings that amino acids other than Leu are involved in the regulation of activity and specificity of Lrp. In place of Leu, Ala has been indicated to act as an effector of Lrp (Berthiaume *et al.*, 2004; Kim *et al.*, 2010; Martin, 1996; Zhi *et al.*, 1998, 1999). A systematic survey of effector function for all amino acids indicated that His, Ile, Met and Thr influence, besides Leu and Ala, Lrp activity (Hart & Blumenthal, 2011). The direction and level of the influence on Lrp activity by each amino acid effector appears variable depending on the target genes and under the culture conditions. The complex nature of Lrp action may be related to its physiological role to harmonize the expression of Lrp regulon genes to match with the surrounding conditions, such as the composition and availability of nutrients.

As a short-cut approach to identify the whole set of regulation target genes of the RNA polymerase (RNAP) sigma subunits and a total of ∼300 species of transcription factors, we developed the Genomic SELEX screening system *in vitro* (Shimada *et al.*, 2005). By using this SELEX system, we succeeded in identifying the whole set of constitutive promoters that are recognized by the RNAP RpoD holoenzyme alone in the absence of supporting transcription factors (Shimada *et al.*, 2014). The functional modulation of RNAP after replacement of sigma factors was then identified by the same SELEX system (T. Shimada and A. Ishihama, in preparation). Along this line, a systematic search of regulation targets by the SELEX system is in progress for ∼300 species of *E. coli* transcription factors. In this study, an attempt was made to identify the regulation target genes that are recognized by Lrp alone in the absence of any effectors. The results herein described indicate a novel role of Lrp in the regulation of a large group of genes involved in not only the transport and metabolism of amino acids, but also the polymerization of amino acids into proteins.

## Methods

### Bacterial strains and plasmids

*E. coli* DH5α was used for plasmid amplification. *E. coli* BL21 was used for Lrp expression. *E. coli* BW25113 (W3110 *lacI*^q^*rrnBT14* Δ*lacZWJ16 hsdR514* Δ*araBADAH33* Δ*rhaBADLD78*) (Datsenko & Wanner, 2000) and JW0872 (a *lrp* single-gene deletion mutant of BW25113) (Baba *et al.*, 2006) were obtained from the *E. coli* Stock Center (National Bio-Resource Center, Mishima, Japan). Cells were grown in M9/glucose medium at 30 °C under aeration with constant shaking at 150 r.p.m. Cell growth was monitored by measuring OD_600_.

### Expression and purification of Lrp

Expression plasmid pLrp of Lrp protein was constructed essentially according to the standard procedure in this laboratory (Shimada *et al.*, 2005; Yamamoto *et al.*, 2005). The Lrp-coding sequence of *E. coli* K-12 W3110 was PCR-amplified and inserted into pET21α between *Nde*I and *Not*I so as to fuse to the C-terminal His-tag. The expression of His-tagged Lrp was performed in *E. coli* BL21. Lrp was affinity-purified according to the standard procedure (Shimada *et al.*, 2005; Yamamoto *et al.*, 2005).

### Preparation of antibodies

Antibodies against Lrp were produced in two rabbits by injecting purified Lrp protein (Ishihama *et al.*, 2014). After examination of antibody activity using immunoblot analysis, the batch of higher activity was used in this study. Antibody production was performed in the Animal Laboratory of Mitsubishi Chemical Medience under the guidelines for animal experiments of the Ministry of Education, Culture, Sports, Science and Technology of Japan.

### Genomic SELEX screening of Lrp-binding sequences

The Genomic SELEX method was carried out as described previously (Shimada *et al.*, 2005). A mixture of DNA fragments of the *E. coli* K-12 W3110 genome was prepared after sonication of purified genome DNA and cloned into a multi-copy plasmid pBR322. In each SELEX screening, the DNA mixture was regenerated by PCR. For SELEX screening, 5 pmol of the mixture of DNA fragments and 10 pmol purified Lrp were mixed in a binding buffer (10 mM Tris/HCl, pH 7.8 at 4 °C, 3 mM magnesium acetate, 150 mM NaCl and 1.25 mg BSA ml^− 1^) and incubated for 30 min at 37 °C. The DNA–Lrp mixture was treated with anti-Lrp antibody, and DNA fragments recovered from the complexes were PCR-amplified and subjected to next cycle of SELEX for enrichment of Lrp-bound DNA fragments.

For SELEX-chip analysis, DNA samples were isolated from the DNA–protein complexes at the final state of SELEX, PCR-amplified and labelled with Cy5, whilst the original DNA library was labelled with Cy3. The fluorescently labelled DNA mixtures were hybridized to a DNA microarray consisting of 43 450 species of 60 bp DNA probes, which were designed to cover the entire *E. coli* genome at 105 bp interval (Oxford Gene Technology) (Shimada *et al.*, 2005, 2008). Fluctuation level of the fluorescent intensity between the 43 450 probes was less than twofold for the original DNA library. The fluorescence intensity of each peak of the test sample was then normalized with that of the corresponding peak of the original library. After normalization of each pattern, the Cy5/Cy3 ratio was measured and plotted along the *E. coli* genome.

### Extraction of metabolites

Samples for intracellular metabolite measurements were processed as described previously (Ohashi *et al.*, 2008; Soga *et al.*, 2003). The exponential-phase culture (OD_600_ 0.5) was filtered under vacuum through a 0.4 μm pore size filter. Cells on the membrane filter were immediately washed with MilliQ water to remove extracellular components and then quickly immersed in 2 ml methanol containing 2.5 μM each of the internal standards, methionine sulfone, MES and d-camphor 10-sulfonic acid. Dishes containing filters were sonicated for 30 s to resuspend the cells. A 1.6 ml aliquot of the cell suspension was transferred to a tube, and mixed with 1.6 ml chloroform and 0.64 ml MilliQ water. After vortexing and centrifugation, the aqueous layer was recovered and clarified using Ultrafree-MC ultrafilter devices for Metabolome Analysis UFC3LCCNB-HMT (Millipore). After drying up, materials attached on the filter were dissolved in 25 μl MilliQ water and subjected to capillary electrophoresis time-of-flight MS (CE-TOF-MS) analysis.

### Instrumentation and CE-TOF-MS conditions

CE-TOF-MS analysis was carried out using an Agilent CE system equipped with an Agilent 6210 TOF mass spectrometer, Agilent 1100 isocratic HPLC pump, Agilent G1603A CE-MS adaptor kit and Agilent G1607A CE-ESI (electrospray ionization)-MS sprayer kit (Agilent Technologies). The system was controlled by Agilent G2201AA ChemStation software for CE. Data acquisition was performed by Analyst QS 7222 software for Agilent TOF (Applied Biosystems and MDSSciex). Instrumental conditions for separations and detections of metabolites were as follows. The cationic metabolites were separated on a fused silica capillary (50 μm × 100 cm) using 1 M formic acid as the electrolyte with the voltage set at 30 kV. A solution of 50  % (v/v) methanol/water was delivered as the sheath liquid at a flow rate of 10 ml min^− 1^ (Soga & Heiger, 2000; Soga *et al.*, 2003). Separations of anionic metabolites and nucleotides were carried out on a COSMO(+)Capillary (Nacalai Tesque) using 50 mM ammonium acetate (pH 8.5) as the electrolyte. The applied voltage was set at − 30 kV. A solution of 5 mM ammonium acetate in 50  % (v/v) methanol/water was delivered as the sheath liquid (Soga *et al.*, 2002; 2003). ESI-TOF-MS was conducted in the positive-ion mode (4000 V) for cationic metabolites, and the negative-ion mode (3500 V) for anionic metabolites and nucleotides. Dry nitrogen gas was maintained at 10 p.s.i. Exact mass data were acquired over a 50–1000 *m*/*z* range (Ohashi *et al.*, 2008; Soga *et al.*, 2006). The raw data obtained using CE-TOF-MS were processed with a proprietary software program, MasterHands, that provided noise-filtering, peak detection and integration of the peaks from sliced electropherograms and alignment of the migration time (Sugimoto *et al.*, 2010). Absolute quantification was performed using metabolite standards for calibration. Under the conditions employed, the deviation of metabolite levels was < 10  % (Soga *et al.*, 2006).

### Phenotype MicroArray (PM) for the growth test

The PM assay was performed essentially according to the published methods (Bochner *et al.*, 2001; Zhou *et al.*, 2003) using Biolog PM plates (Biolog). *E. coli* BW25113 and JW0872 were grown overnight at 30 °C in M9/glucose (0.2  %). Cells were washed with IF-0 (inoculating fluid), and then resuspended in IF-0 for PM plates 1 and 2, in IF-0 containing 20 mM sodium succinate and 2 mM ferric citrate for PM plates 3–8, and in IF-10 containing 2.0 g tryptone, 1.0 g yeast extract and 1.0 g NaCl l^–1^ at a density corresponding to 85  % transmittance (OD_420_∼0.12) using a 20 mm diameter tube. Tetrazolium violet was added at the final concentration of 0.01  %. The suspensions were then inoculated into the appropriate microplates PM1–10 for bacteria (Biolog) at a volume of 0.1 ml per well. The microplates were placed in an OmniLog instrument at 30 °C and monitored by OmniLog reader (Biolog) for colour change in the wells at 15 min intervals up to 72 h. Kinetic data were analysed with OmniLog-PM software. Each strain was tested at least twice.

### Northern blot analysis

Total RNAs were extracted from exponentially growing *E. coli* cells (OD_600_ 0.5) by the hot phenol method. RNA purity was checked by electrophoresis on 1.5  % agarose gel in the presence of formaldehyde followed by staining with methylene blue. Northern blot analysis was performed essentially as described previously (Shimada *et al.*, 2007, 2011). DIG-labelled probes were prepared by PCR amplification using W3110 genomic DNA (50 ng) as template, DIG-11-dUTP (Roche) and dNTP as substrates, gene-specific forward and reverse primers, and Ex *Taq* DNA polymerase (TaKaRa). Total RNAs (1 mg) were incubated in formaldehyde-MOPS gel-loading buffer for 10 min at 65 °C for denaturation, subjected to electrophoresis on formaldehyde-containing 1.5  % agarose gel and then transferred to a nylon membrane (Roche). Hybridization was performed with a DIG easy Hyb system (Roche) at 50 °C overnight with a DIG-labelled probe. For detection of the DIG-labelled probe, the membrane was treated with anti-DIG-AP Fab fragments and CDP-Star (Roche), and the image was scanned with a LAS-4000 IR multi-colour imager (Fuji Film).

## Results

### Search for Lrp-binding sequences by Genomic SELEX screening

**Fig. 1.**
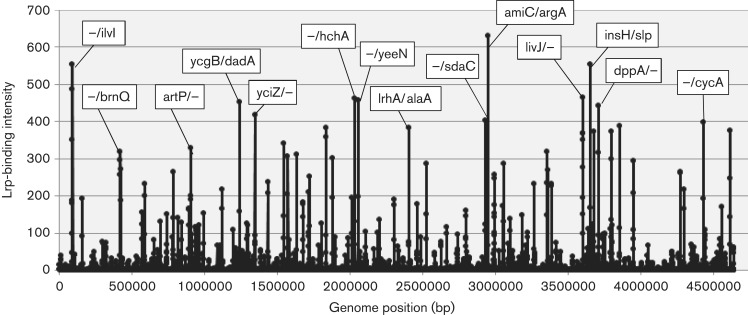
Lrp-binding sites on the *E. coli* K-12 genome identified by SELEX-chip. After two cycles of Genomic SELEX screening, a collection of Lrp-bound DNA fragments was subjected to SELEX-chip analysis using the tilling array of the *E. coli* K-12 genome (for details, see Methods). The *y*-axis represents the relative number of Lrp-bound DNA fragments, whereas the *x*-axis represents the position on the *E. coli* genome. The regulation targets were predicted based on the location of Lrp-binding sites. For Lrp sites within type A spacers, both of the flanking genes of the bidirectional transcription units are shown. For Lrp sites within type B spacers, only the genes located downstream of the Lrp sites are shown, but the genes on the other side are shown as a minus symbol. Details of target genes are listed in Table S1.

In order to identify the whole set of target promoters, genes and operons under the direct control of Lrp, we performed Genomic SELEX screening (Shimada *et al.*, 2005), in which purified His-tagged Lrp was mixed with a collection of *E. coli* genome fragments of 200–300 bp in length and Lrp-bound DNA fragments were affinity-isolated. As the specificity of target recognition of Lrp is known to change toward different directions, depending on the species of interacting amino acid effector (Hart & Blumenthal, 2011), in this study we carried out SELEX screening using 0.1 μM Lrp alone in the absence of effectors. Under these conditions, Lrp exists mainly in the monomer state as estimated from the known association constants, but a possible influence of the C-terminal His-tag addition on its multimerization is not ruled out. The list of DNA sequences thus identified should provide the basic set of regulation targets by Lrp alone. The original mixture of genomic DNA fragments formed smear bands on PAGE, but after two cycles of Genomic SELEX, DNA fragments with high affinity to Lrp were enriched, forming sharper bands on PAGE gels (data not shown). As a short-cut approach to identify the whole set of sequences recognized by Lrp, we subjected this isolated SELEX fragment mixture to DNA chip analysis using an *E. coli* tilling array (Shimada *et al.*, 2008, 2011). In brief, the SELEX DNA fragments were labelled with Cy5 whilst the original DNA library was labelled with Cy3. The mixtures were then hybridized with the DNA tilling microarray (Oxford Gene Technology) and the fluorescence intensities bound on each probe were measured. For identification of Lrp-binding sites, the Cy5/Cy3 ratio was plotted along a total of 43 450 probes aligned on the array in the order of the *E. coli* genome (Fig. 1).

By setting a cut-off level of the Genomic SELEX pattern at 10 (Fig. 1), a total of 314 Lrp-binding peaks were identified, of which 228 (72  %) were within intergenic spacers and 86 (28  %) were inside ORF regions (Table 1). The Lrp-binding spacers could be classified into three groups: type A, spacers between bidirectional transcription units (78 spacers); type B, spacers upstream of one transcription unit, but downstream of another transcription unit (140 spacers); and type C (10 spacers), spacers downstream of both transcription units (Table 1). In the case of type A spacers, Lrp might regulate one or both of the transcription units, whilst Lrp bound within type B spacers should be involved in regulation of one-directional transcription. Up to the present time, we have performed SELEX-chip screening for >150 *E. coli* transcription factors (for a review, see Ishihama, 2012); some, but not always, showed binding within type C spacers, implying an as-yet unidentified regulatory role for this group of transcription factor binding. Likewise, the total of 86 Lrp-binding sites inside ORFs may play certain regulatory roles because the amount of transcription factor-binding sites inside ORFs varies depending on transcription factor species (Ishihama, 2012; Shimada *et al.*, 2008).

**Table 1.** SELEX-chip screening of Lrp-binding sequences: Lrp-binding sites on the *E. coli* genome. A total of 314 Lrp-binding sites can be classified into three groups: type A, spacers between bidirectional transcription units (78 spacers); type B, spacers upstream of one transcription unit but downstream of another transcription unit (140 spacers); and type C (10 spacers), spacers downstream of both transcription units.

Location	No. Lrp sites	No. targets	RegulonDB	ChIP-chip
Within type A spacers	78	78–156	9	32
Within type B spacers	140	140	15	55
Within type C spacers	10	0	0	0
Inside ORFs	86	(89)	0	0
	314	218–296	24	87

### Prediction of the regulation targets of Lrp

In prokaryotes, transcription factors generally bind near the promoter for effective interaction with promoter-bound RNA polymerase, and thus the target genes and promoters under the control of Lrp could be estimated based for the Lrp-binding sites within type A and type B spacers. Based on the location of Lrp-binding sites on the *E. coli* genome, we then predicted the set of regulation target genes and operons recognized by Lrp alone. The total number of Lrp regulation targets thus estimated ranged between a minimum of 218 (type A 78 plus type B 140) and a maximum of 296 (type A 156 plus type B 140) (Table 1; for details see Table S1, available in the online Supplementary Material). The total number of regulation targets of Lrp has been estimated to be ∼130 based on ChIP-chip analysis (Cho *et al.*, 2008) whilst the number of Lrp targets listed in RegulonDB is 43 (Salgado *et al.*, 2006). The list of regulation targets predicted based on the SELEX screening covered 87 (67  %) of ChIP-chip data and 24 (60  %) of the RegulonDB list (Table 1). In order to avoid background noise, we set a rather high cut-off level at 10 (see Fig. 1) and, as a result, we failed to pick up some of the known targets, of which most could be recovered by setting the cut-off level at 3.0 (data not shown).

The total number of Lrp targets increased ∼2.3-fold from 130 up to 296. The marked increase in the number of regulation targets has been identified for not only Lrp, but also most of the transcription factors so far examined by SELEX screening (Ishihama, 2010, 2012; Shimada *et al.*, 2011). This increase was mainly attributable to the difference between *in vitro* estimation by SELEX and *in vivo* measurement by ChIP-chip. The binding *in vivo* of Lrp should be interfered by competitive binding by other DNA-binding proteins. In addition, the intracellular conditions were different from *in vitro* SELEX conditions, altogether influencing the Lrp–DNA interaction modes. Amongst the total of 296 candidate genes under the direct control of Lrp, 114 were related to the metabolism of amino acids (Table 3, type A plus type B lane). This value corresponded to 89  % of the hitherto identified genes involved in the synthesis and degradation of amino acids, in good agreement with the predicted regulatory functions of Lrp. A total of 261 transporter genes, including 43 transporters of amino acids, are listed in Genobase. After SELEX screening, a total of 84 transporter genes were found to be under the direct control of Lrp (Table 3, type A plus type B lane), of which 35 represented the genes for amino acid transporters (80  % of total amino acid transporters) (Table 2).

**Table 2.** SELEX-chip screening of Lrp-binding sequences: Lrp regulon genes involved in transport and metabolism of amino acids The Lrp regulon genes involved in transport and metabolism of amino acids, tRNA, tRNA charging and rRNA are listed. The number of the whole set of genes involved in those functions is shown in Whole set column. The number of genes identified by SELEX screening is shown in the SELEX-chip column. The number of genes listed in RegulonDB (Salgado *et al.*, 2006) or ChIP-chip analysis (Cho *et al.*, 2008) is shown in the DB+ChIP-chip columns. Percentage shows the coverage of the whole set of genes.

Function	Whole set	SELEX-chip (%)	DB+ChIP-chip (%)
Transporter	43	35 (81)	24 (56)
Metabolism	128	114 (89)	41 (32)
tRNA	85	17 (20)	10 (12)
tRNA charging	24	6 (25)	1 (4)
rRNA	21	9 (43)	21 (100)

**Table 3.** Lrp-binding sites on the *E. coli* genome A total of 314 Lrp-binding sites were identified within spacers on the entire *E. coli* K-12 W3110 genome. A total of 78 Lrp-binding sites were identified within type A spacers, which direct bidirectional transcription. A total of 140 Lrp-binding sites were located within type B spacers upstream of one-side genes and downstream of another-side genes. Based on the gene orientation around these binding sites, the genes and operons under the control of Lrp were estimated. Lrp-binding sites listed in RegulonDB (Salgado *et al.*, 2006) or ChIP-chip analysis (Cho *et al.*, 2008) are shown in the DB or ChIP-chip columns. Genes encoding amino acid metabolism, translation apparatus, transporters and transcription factors are shown in AA, TR, TP and TF columns, respectively.

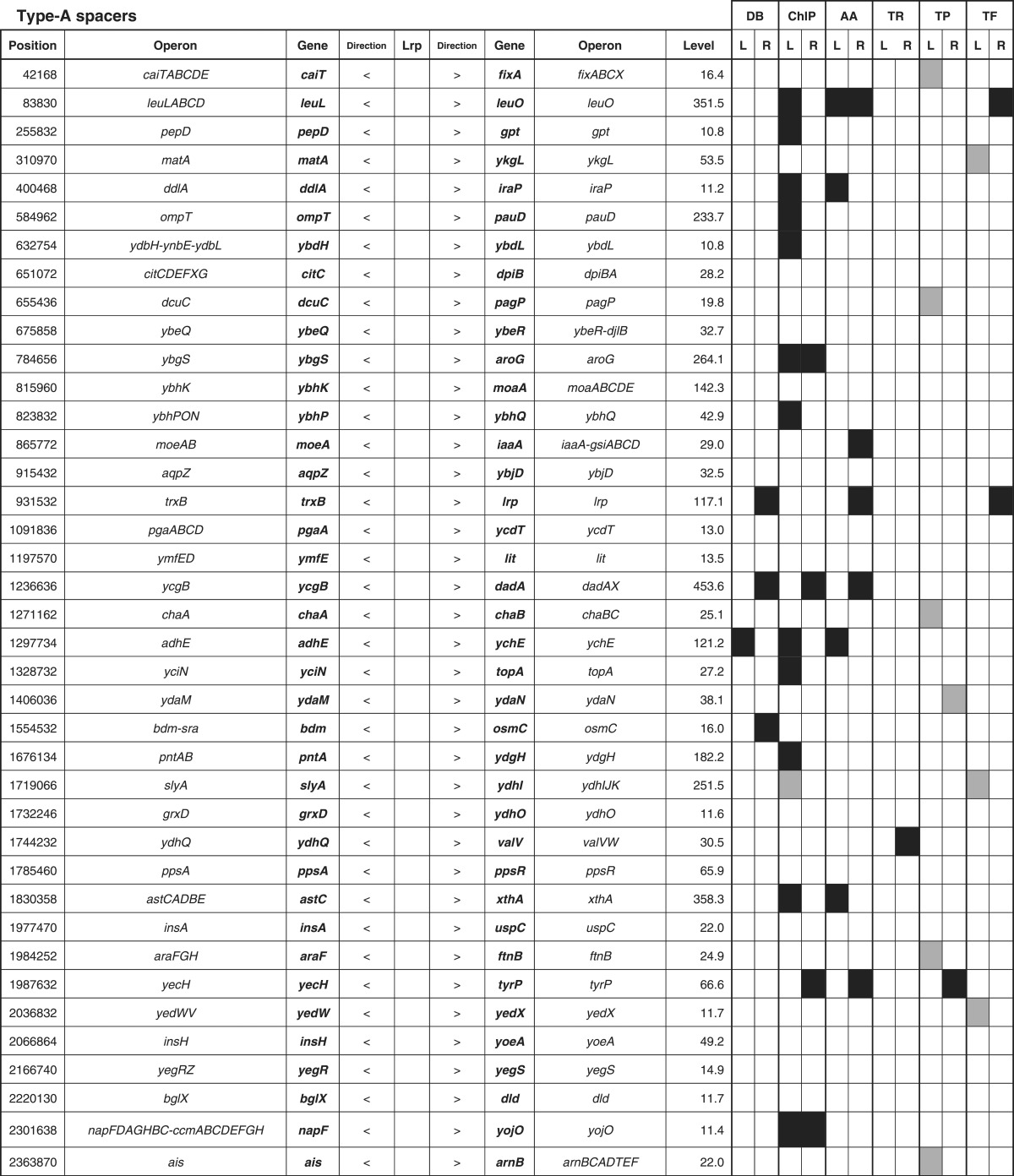																				
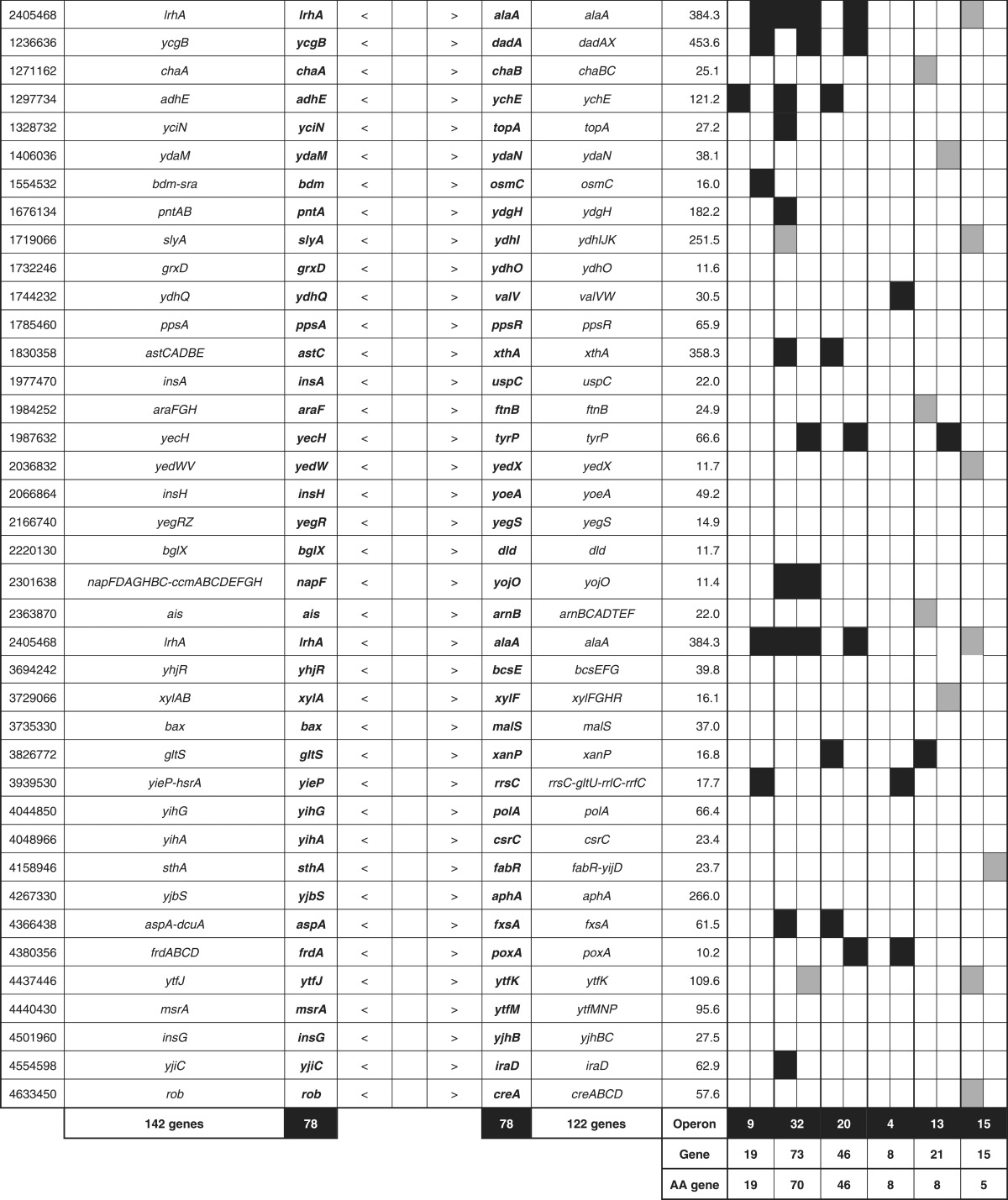																				
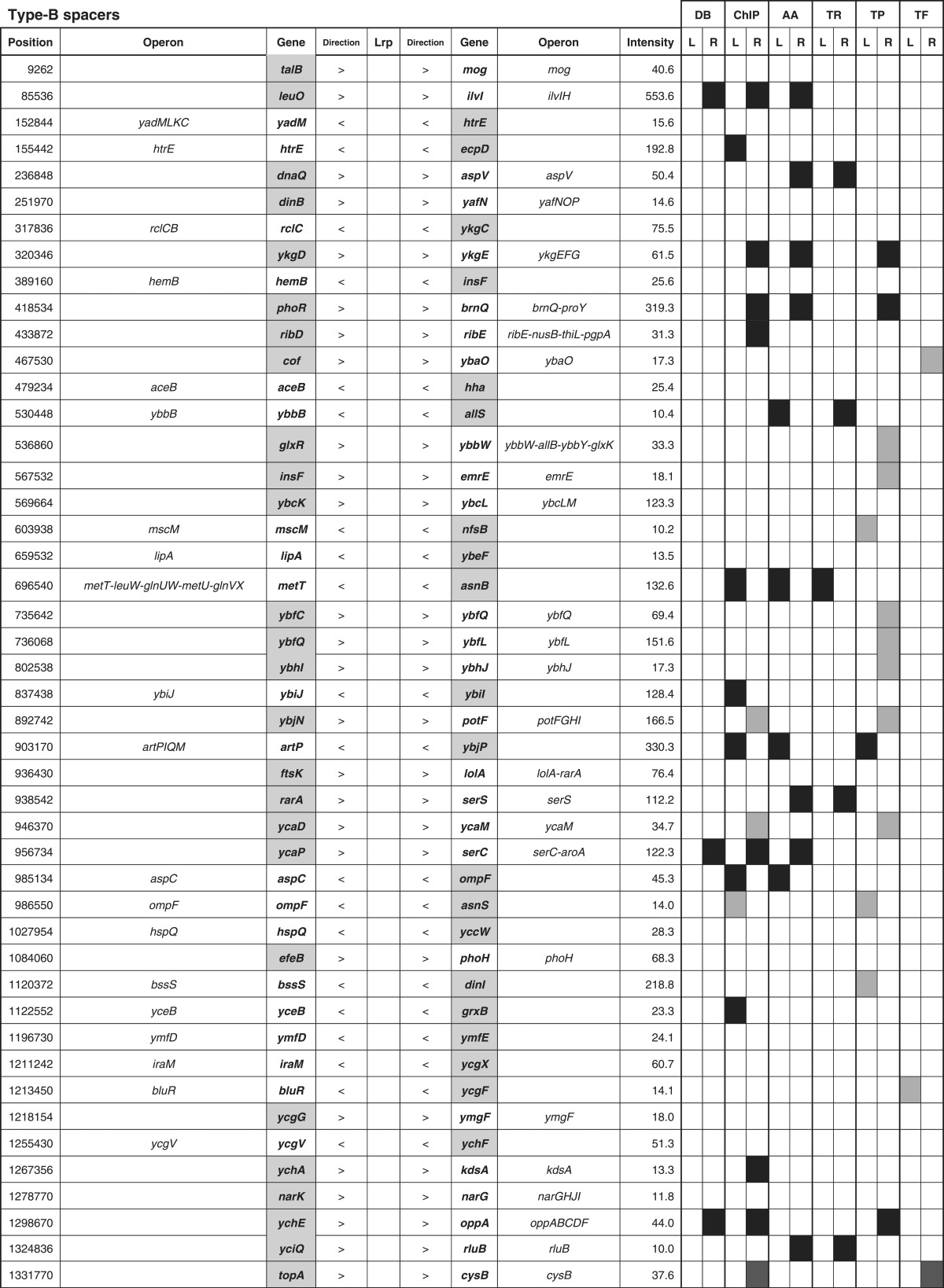																				
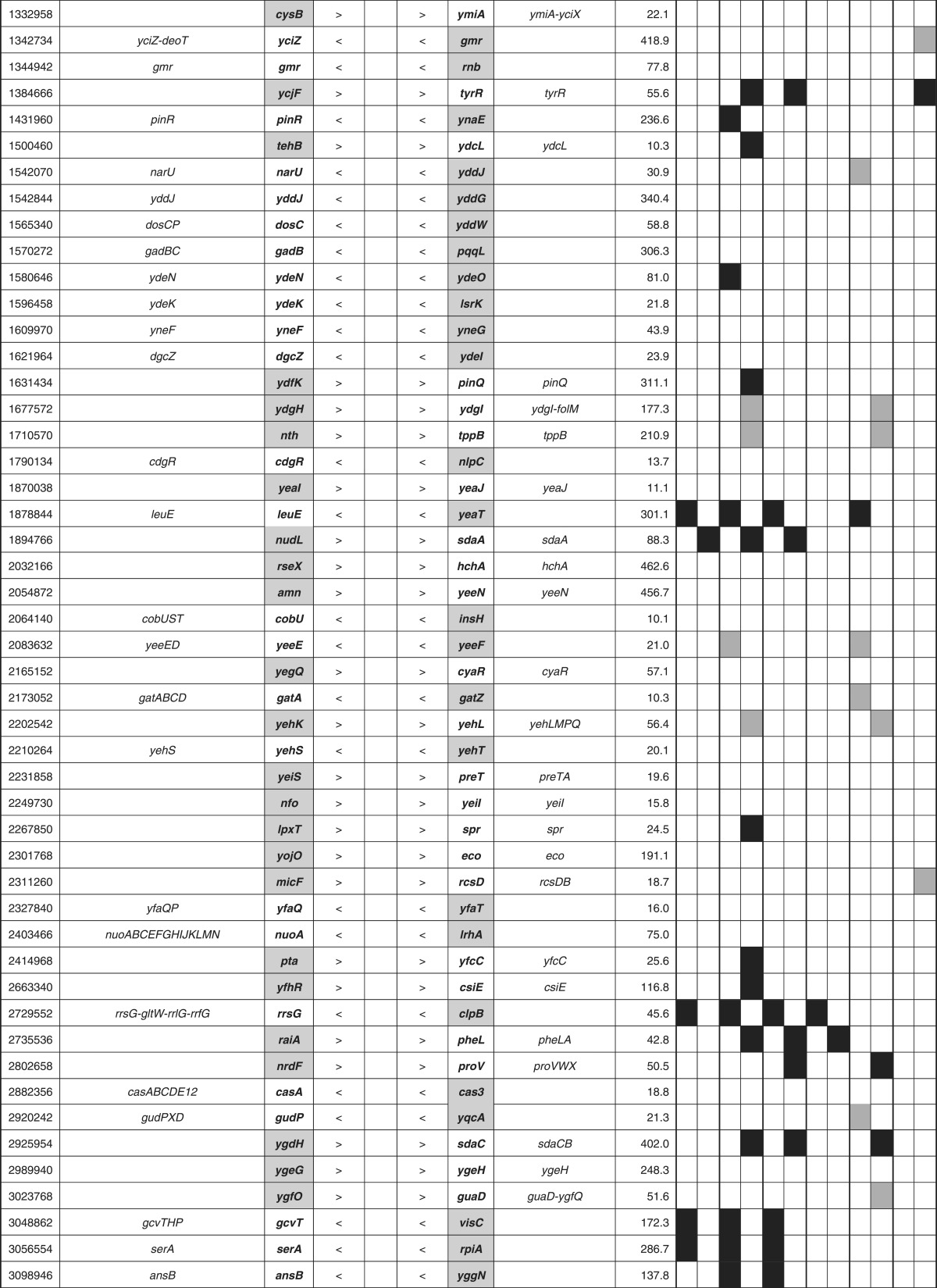																				
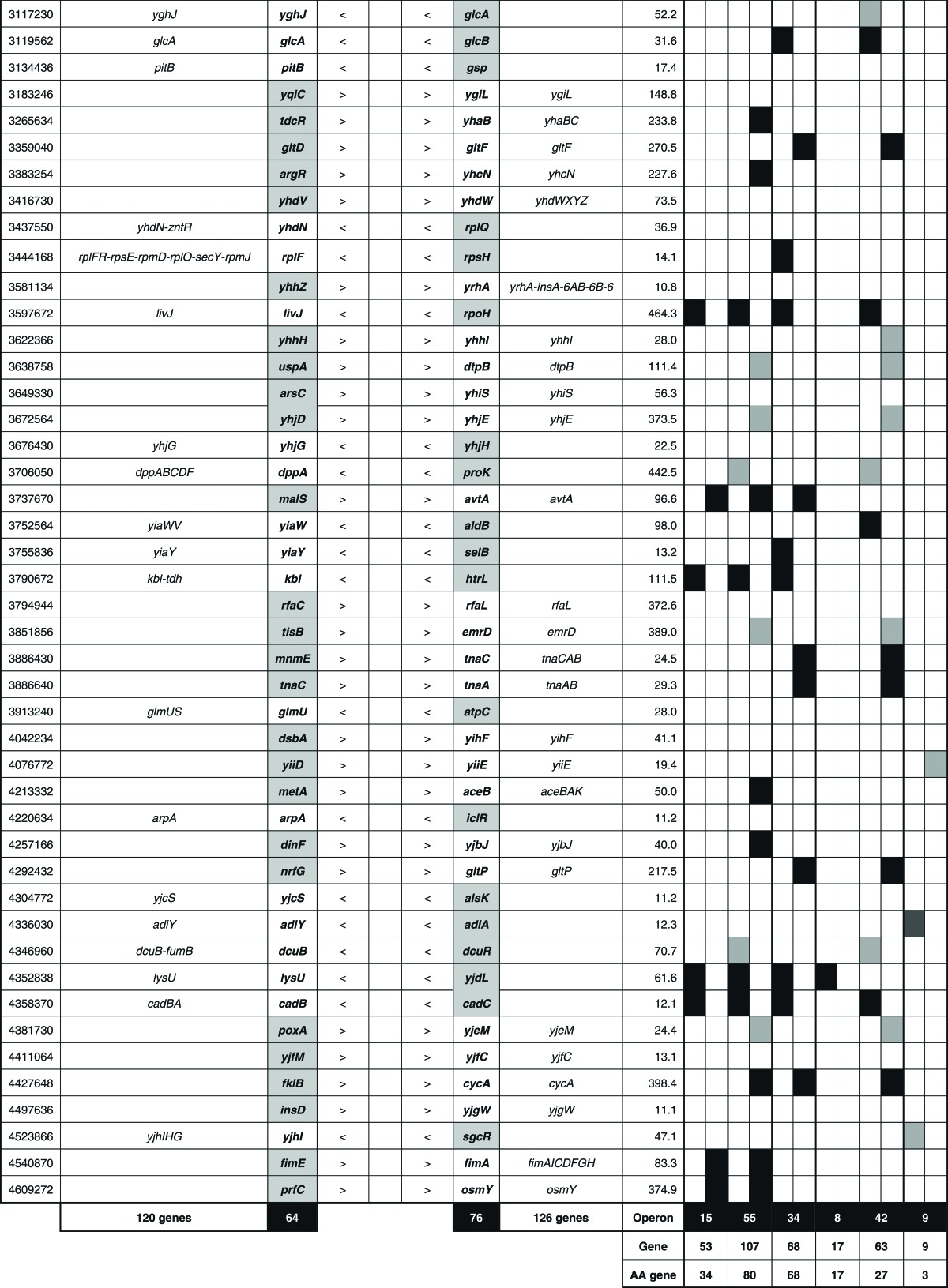																				
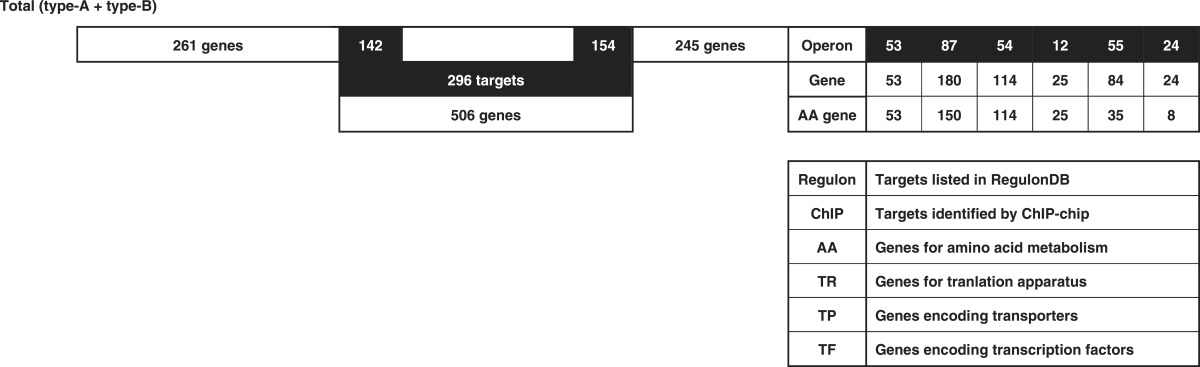																				

### Search for the regulatory roles of Lrp: PM

**Fig. 2.**
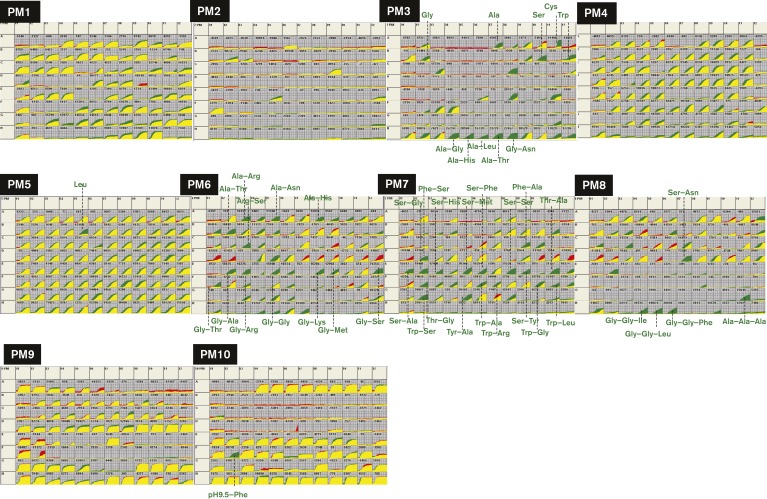
PM analysis of the Lrp mutant. PM analysis of *E. coli* WT BW25113 and its *lrp* mutant JW0872 was performed using the Biolog PM apparatus according to the procedure provided by the provider. Growth patterns of microplates PM1–10 are shown: PM1 and 2, carbon source metabolism; PM3, nitrogen source metabolism; PM4, phosphorus and sulfur source metabolism; PM5, nutrient supplements; PM6–8, peptide as nitrogen metabolism; PM9, osmotic and ion effects; PM10, pH effects. The curve of each well shows the time-course (*x*-axis, up to 3 days) of cell growth as determined by measuring the amount of purple colour (*y*-axis) formed from tetrazolium dye reduction. Data from the WT strain are shown in green, whilst data from the *lrp* mutant are shown in red. Yellow shows the overlap of the two growth curves. Details are listed in Table S2.

After Genomic SELEX screening, we recognized a sudden and marked increase in the list of regulation targets of Lrp, indicating that Lrp plays as-yet unidentified regulatory roles in overall transcription of the *E. coli* genome. As an attempt to obtain insights into the regulatory role of Lrp, we performed a PM assay, which allows the detection of cell growth under a total of 960 culture conditions: the presence of 192 species of carbon source (PM plates 1 and 2), 96 species of nitrogen source (PM plate 3), 96 species of phosphorus and sulfur sources (PM plate 4), 96 species of nutrient supplement (PM plate 5), 288 chemicals as peptide nitrogen source (PM plates 6–8), 96 species of osmolyte (PM plate 9) and 96 different pH conditions (PM plate 10) (Bochner, 2009). We measured the growth of WT *E. coli* BW25113 and JW0872 (*lrp* single-gene deletion mutant of BW25113). The time-course of cell growth was monitored by measuring the cell density-dependent increase in respiration (Bochner *et al.*, 2001). After 3 days of culture, the difference of growth between the WT and the *lrp* mutant was estimated by comparison of the growth curves (Fig. 2). Growth rates of the WT and *lrp* mutant were essentially the same in the absence of any additions (see microplate well 1 for each PM plate).

The *lrp* mutant strain exhibited slower growth under a total of 59 conditions, of which 50 were in the presence of specific nitrogen sources, four in the presence of specific carbon sources, four in the presence of nutrient supplement and one at specific pH (marked in green for representative compounds in Fig. 2). It is noteworthy that the *lrp* mutant showed significantly reduced growth especially in the presence of Ala, Cys, Gly, Ser and Trp as a sole nitrogen source (Fig. 2, PM plate 3; for details, see Table S2) and some peptides such as Ala–Gly, Ala–Leu, Gly–Asn, Ala–His and Ala–Thr, each including one of these five amino acids (Fig. 2, indicated in green colour; see Table S2 for the entire list). Reduction of *lrp* mutant growth in the presence of Ser agrees with the previous observation (Ambartsoumian *et al.*, 1994). In contrast, growth of the *lrp* mutant was slightly enhanced in the presence of dipeptides including Asp, Glu and Pro as a sole nitrogen source (Fig. 2, shown in red and Table S2). These results suggested that the function of Lrp was needed for utilization of some of these specific amino acids as sole nitrogen sources. In the simultaneous presence of NH_4_Cl, the addition of amino acids did not affect growth of the *lrp* mutant (Fig. 2, plate 5). One exception was the culture in the presence of both NH_4_Cl and Leu, in which growth of the *lrp* mutant was significantly reduced, indicating that excess of Leu specifically interferes with cell growth in the absence of Lrp.

### Search for the physiological role of Lrp: metabolome analysis

**Fig. 3.**
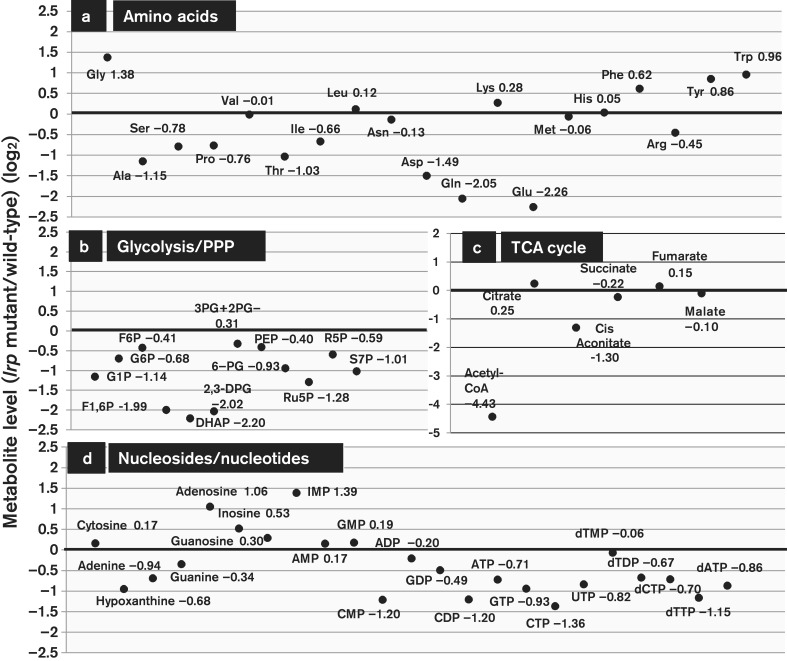
Difference of intermediate metabolites between WT and *lrp* mutant. *E. coli* WT BW25113 and its *lrp* mutant JW0872 were cultured in M9/0.2  % glucose medium until OD_600_ 0.2 and all the intracellular metabolites were extracted as described in Methods. The samples were subjected to CE-TOF-MS analysis according to the standard procedures as described in Methods. The intermediate metabolites are classified into amino acids (a), intermediate metabolites of the glycolysis/pentose phosphate pathway (PPP) (b), metabolites in the tricarboxylic acid (TCA) cycle (c) and nucleosides/nucleotides (d). The ratio of metabolite levels between WT and the *lrp* mutant (*y*-axis) is shown by log_2_.The level of difference of each metabolite is shown in Fig. 4 and details of the measurements are described in Table S3.

**Fig. 4.**
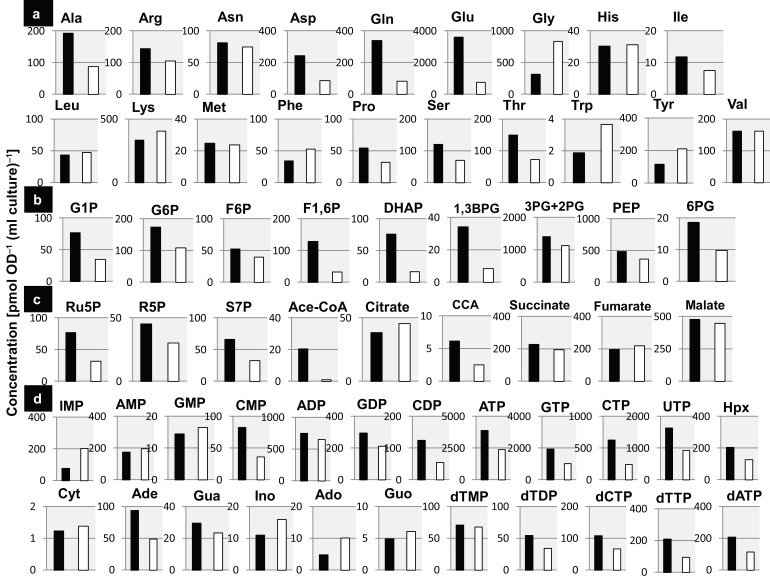
Intracellular concentrations of major metabolites in WT and the *lrp* mutant. The intracellular concentrations of metabolites in WT BW25113 (black bar) and its *lrp* mutant JW0872 (white bar) were determined by CE-TOF-MS. Major metabolisms that showed different concentrations between the two strains are shown. Classification of the metabolites is as in Fig. 3.

Results of the PM analysis indicated that the intracellular composition of metabolites might be altered in the absence of Lrp. To test this prediction, we next carried out the metabolome analysis using CE-TOF-MS. For the cells grown in M9/glucose medium, a set of metabolites was measured for both the WT and *lrp* mutant strains. The overall metabolite profiles indicated a considerable variation in the intracellular concentrations of not only amino acids, but also some intermediate metabolites in the glycolysis/pentose phosphate pathways and tricarboxylic acid cycle (Fig. 3; for each metabolite see Fig. 4 and Table S3). The level of Gly, Phe, Tyr and Trp was markedly higher in the *lrp* mutant. In contrast, the level of Glu, Gln and Asp was lower in the *lrp* mutant. The changes in amino acid levels might be due to the regulation network of transcription factors for control of amino acid synthesis and utilization. For instance, the highly accumulated aromatic amino acids are all under the control of a single transcription factor, TyrR (regulator of aromatic amino acid synthesis). Likewise, transcription factors of the genes for amino acid metabolism, including AdiY and CysB (regulator of Cys synthesis), LeuO (regulator of Leu synthesis), and TdcA and TdcR (regulator of Thr synthesis), are all under the direct control of Lrp (Tables 3 and S1) and thus the expression of a number of genes involved in the metabolism of amino acids should be indirectly regulated in the absence of Lrp, leading to influence in the intracellular pool of respective amino acids.

The change in amino acid levels was interconnected with the changes in the level of intermediate metabolites of carbohydrate catabolism and energy metabolism. Some specific amino acids showed a reverse correlation between the influence on cell growth and the intercellular concentration. In the presence of some dipeptides, such as Glu and Pro, as a sole nitrogen source, the *lrp* mutant showed a higher rate of cell growth than the WT cells. The intracellular concentrations of Glu and Pro in the *lrp* mutant were lower than those in WT (compare Figs 2 and 3). These growth and metabolic behaviours indicate that effective availability of Glu and Pro in the *lrp* mutant cells resulted in the promotion of growth. However, in the presence of some other dipeptides, such as Gly and Trp, as a sole nitrogen source, the growth of the *lrp* mutant was slower than the WT and their intracellular concentrations were higher than the WT. The lower availability of these amino acids in the *lrp* mutant resulted in growth retardation and accumulation of amino acids. This reverse correlation implies that a group of amino acids closely linked to the metabolic pathways for the production of metabolic energy is preferentially utilized for the high growth rate of the *lrp* mutant, thereby showing decreased levels of their intracellular pools.

In the absence of Lrp, a marked change was also observed in the intracellular composition of not only amino acids, but also other metabolites (Fig. 3b–d). In particular, a marked difference was detected in the level of acetyl-CoA, a major source of the metabolic energy, and the key player in the degradation and synthesis of lipids and amino acids. The level of acetyl-CoA in the *lrp* mutant was 25-fold less than that in the WT cells (Fig. 4c). Likewise, the level of dihydroxyacetone phosphate, 1,3-bisphosphoglycerate, fructose 1,6-diphosphate, the intermediates of glycolysis, was lower in the *lrp* mutant (Fig. 4b). The observed metabolic changes support the prediction of the coordinated linkage of carbon metabolism with the alteration of amino acid metabolism. The reduction of CMP, CDP, CTP, GTP and UTP (Figs 3 and 4) might lead to the decrease in RNA synthesis in the *lrp* mutant.

### Transcription regulation of the newly identified targets by Lrp

Results of the SELEX-chip screening supported the concept that Lrp is a global transcription regulator for the set of genes involved in transport, synthesis and degradation of amino acids. The results of the PM assay and metabolome analyses are both consistent with this concept. In addition, Lrp was found to be involved in regulation of the genes for the utilization of amino acids in the pathway of translation, such as tRNA, tRNA aminoacylation, rRNA and ribosomal proteins (Table 3; for details, see Table S1). *E. coli* carries a total of 23 genes for aminoacyl-tRNA synthetase. Up to the present time, regulation by Lrp has been recognized only for the *lysU* gene that encodes lysyl-tRNA synthetase (Gazeau *et al.*, 1992), but no transcriptional regulators have been identified for the other 22 aminoacyl-tRNA synthetase genes [note that both GlyRS and PheRS are composed of two different subunits, and *E. coli* contains two forms (constitutive and inducible) of LysRS]. After the Genomic SELEX screening, Lrp was found to bind the promoter region of at least eight aminoacyl-tRNA synthetase genes (*alaS*, *asnS*, *glnS*, *glyQ*, *pheS*, *serS*, *thrS* and *tyrS*), implying the involvement of Lrp in transcription regulation of these genes.

**Fig. 5.**
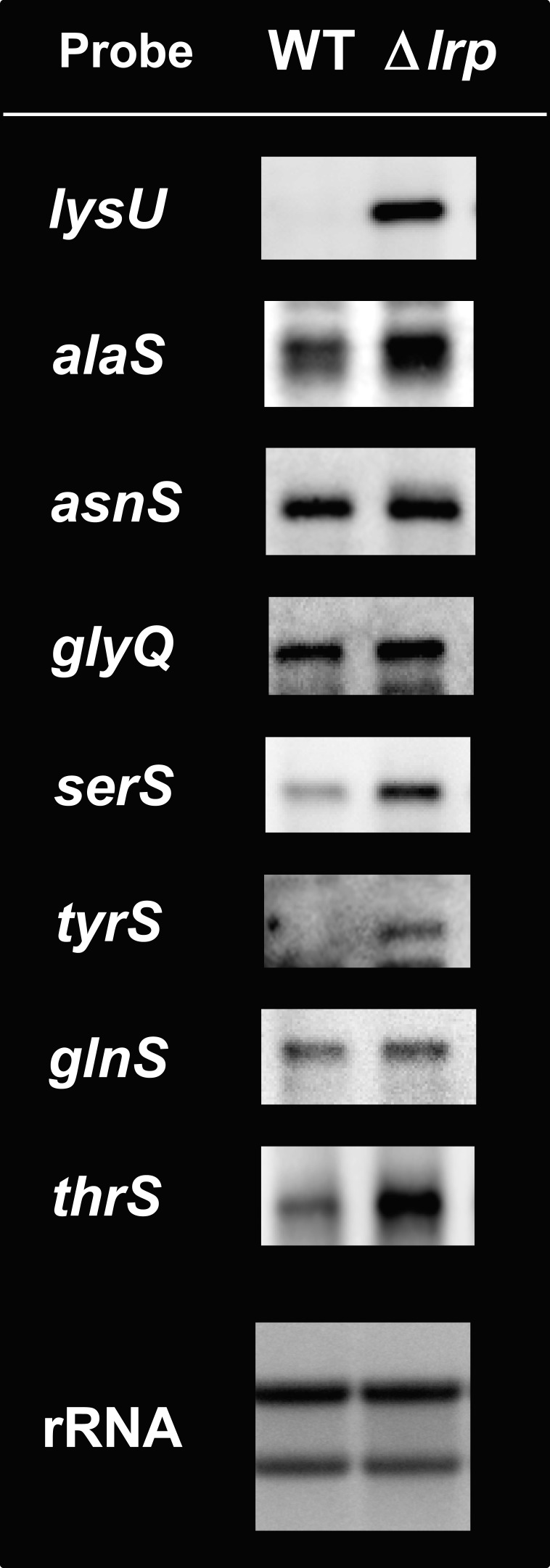
Northern blot analysis of aminoacyl-tRNA synthetase mRNAs. WT BW25113 and its *lrp* mutant JW0872 were grown in M9/0.2  % glucose medium. Total RNA was prepared at the exponential phase and directly subjected to Northern blot analysis under the standard conditions as described in Methods. DIG-labelled hybridization probes are shown on the left side of each panel. The amounts of total RNA analysed were calculated by measuring the levels of 23S and 16S rRNAs stained with methylene blue.

In order to examine regulation *in vivo* of these aminoacyl-tRNA synthetase genes by Lrp, we performed Northern blot analysis for detection of mRNA from these genes. RNA samples were prepared from both *E. coli* WT BW25113 and the *lrp* mutant JW0872, and subjected to Northern blot analysis (Fig. 5). mRNA of *lysU*, the known target of Lrp, was virtually undetectable in the WT strain under the culture conditions employed, but a high level of *lysU* mRNA was detected in the *lrp* mutant strain, indicating strong repression of the *lysU* gene by Lrp. Next, we analysed the level of mRNAs for seven other aminoacyl-tRNA genes. The levels of *serS*, *tyrS* and *thrS* were low in WT cells, but increased in the *lrp* mutant, as in the case of *lysU*. mRNAs of other aminoacyl-tRNA genes were detected even in WT cells, but *alaS* mRNAs increased, albeit at low levels, in mutant cells. Thus, we concluded that Lrp participates in transcription regulation of at least eight aminoacyl-tRNA synthetase genes, of which expression of five aminoacyl-tRNA synthetase, including AlaRS, LysRS, SerRS, ThrRS and TyrRS, is repressed by Lrp. So far only minimal Lrp-dependent changes have been observed in the microarray analysis (Tani *et al.*, 2002), which was, however, carried out in the cultures in the presence of Ile and Val addition. In general, Northern blot analysis gives a more accurate estimation of individual mRNA than microarray analysis.

## Discussion

### Regulatory roles of Lrp

After SELEX-chip screening, at least 296 regulation targets were identified for Lrp, resulting in an increase of ∼2.3-fold. One group of the novel targets includes the genes for utilization of amino acids such as the genes encoding tRNA, aminoacyl-tRNA synthetase, rRNA and ribosomal proteins. Here, a total of eight aminoacyl-tRNA synthetase genes were identified to be under the direct control of Lrp, but this number increases by setting the cut-off level of SELEX pattern < 10 (Fig. 1). In the case of rRNA operons, all seven rRNA operons have been reported to be under the control of Lrp (Pul *et al.*, 2005). In this study, only three were identified by setting the cut-off level at 10, but all seven known rRNA operons could be identified by setting the cut-off level at 3.0. The whole set of regulation targets herein identified indicates that Lrp senses the presence of nutritional conditions and regulates not only the transport and metabolism (synthesis and degradation) of amino acids, but also the utilization amino acids up to protein synthesis. It should be noted, however, that the selectivity of regulation targets by Lrp should be altered after interaction of an effector ligand.

*E. coli* contains as many as 300 species of transcription factors, each monitoring a specific factor or condition in the environment (Ishihama, 2010, 2012). The majority of *E. coli* transcription factors belong to the one-component signal transduction system, in which a single polypeptide contains both an effector-binding sensory domain and a DNA-binding domain. The activity of this group of transcription factors is controlled by a single species of the effector ligand, i.e. inducer or co-repressor. In some cases, the involvement of two effectors has been identified: allantoin and glyoxalate for AllR (Hasegawa *et al.*, 2008), Arg and Lys for ArgP (Marbaniang & Gowrishankar, 2011), glyoxylate and pyruvate for IclR (Lorca *et al.*, 2007), hypoxanthine and guanine for PurR (Houlberg & Jensen, 1983), and uracil and thymine for RutR (Shimada *et al.*, 2007). Moreover, t activity control by more than three effectors has been recognized recently for a set of transcription factors such as CueR by Cu(II), Ag(II) and Au(II) (Ibanez *et al.*, 2013), TyrR by Tyr, Trp and Phe (Pittard, 1996), and SdiA by three HSL analogues (Shimada *et al.*, 2013). In this respect, Lrp is unique because its function is considered to be regulated at various levels by not only Leu, but also Ala, His, Ile, Met and Thr (Hart & Blumenthal, 2011). The next step in the research is to identify the whole set of regulation targets of Lrp in the presence of each effector ligand.

### Hierarchy of the transcription factor network involving Lrp

**Fig. 6.**
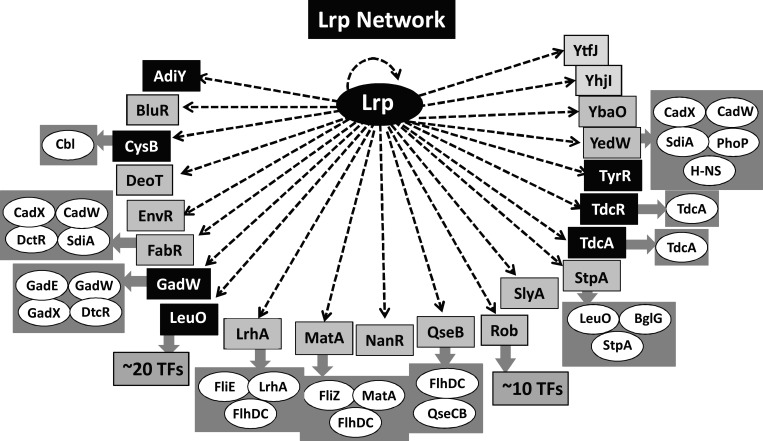
Network of transcription factors (TFs) involving Lrp. After SELEX-chip screening, a total of 23 transcription factors were indicated to be under the direct control of Lrp, altogether forming a big network, in which Lrp is located on the top of the hierarchy. In addition, the genes encoding other transcription factors are organized downstream of some of these transcription factors.

In the collection of a total of 296 Lrp targets selected by SELEX-chip screening, a set of 21 transcription factor genes was identified, including the *lrp* gene itself (Fig. 6). Interestingly, the genes coding for local regulators of the genes for individual amino acids are under the control of Lrp, including AdiY (a regulator of Arg regulon), CysB (a regulator of the Cys regulon), GadW (a regulator of Glu regulon), LeuO (a regulator of Leu regulon), TdcA and TdcR (regulators of the Thr and Ser regulons), and TyrR (a regulator of Tyr regulon) (Fig. 6, filled symbols). AdiY and GadW are also involved in expression of the low pH response genes for control of intracellular pH by using Arg and Glu, respectively (Ma *et al.*, 2002; Stim-Herndon *et al.*, 1996). LeuO is another Leu-sensing global regulator that controls ∼140 targets, of which most are involved in anti-silencing against the H-NS silencer (Shimada *et al.*, 2011).

In addition to these amino acid-related transcription factors, Lrp was found to regulate a total of 15 transcription factors (Fig. 6, grey symbols), which are involved in the stress-response and life-style selection of *E. coli*, such as GadW for acid response, YedW for copper and peroxide response, EnvR for response to drugs, QseB for quorum sensing, BluR for biofilm formation, and MatA and SlyA for planktonic growth. Thus, the life style of *E. coli* under these transcription factors is also under the control of Lrp, which monitors the nutritional conditions in the environment. Lrp is located upstream of this hierarchic network including these two groups of transcription factors, thereby regulating a large number of genes indirectly besides the total of ∼300 direct targets.

## Supplementary Data

437 Supplementary Data
