# Supplementary material for: Expanded roles of leucine-responsive regulatory protein in transcription regulation of the Escherichia coli genome: Genomic SELEX screening of the regulation targets
Source: Microb Genom. 2015 Jul 15;1(1):e000001. doi: 10.1099/mgen.0.000001 (PMC5320599; doi:10.1099/mgen.0.000001)
Supplement: Supplementary file 1 — Supplementary Data [file mgen-01-1-s001.pdf]

Supplementary data

Journal name: MICROBIAL GENOMICS

Article title: Expanded roles of Lrp in transcription regulation of the *Eschericia coli* genome: Genomic SELEX screening of the regulation targets

Author names: Tomohiro Shimada, Natsumi Saito, Michihisa Maeda, Kan Tanaka and Akira Ishihama

Affiliation of the corresponding author: Chemical Resources Laboratory, Tokyo Institute of Technology, Nagatsuda, Yokohama, Japan

Email address of the corresponding author: tshimada@res.titech.ac.jp

This article has 3 supplemental tables.

Supplemental Table S1. Lrp-binding sites on the *E. coli* genome. Genomic SELEX screening of Lrp-binding sequences were identified in a total of 314 sites on the *E. coli* genome (see Table 1). When Lrp-binding sites are located within spacer regions, only the flanking genes are indicated. The direction of transcription is shown by arrows. The genes shown in parentheses carry the Lrp-binding sites on the open reading frames. These Lrp-binding sites are classified into four groups: Type-A spacer; type-B spacer; type-C spacer; and inside open reading frames. Type-A and type-B spacers and their flanking genes are listed in this table.

Supplemental Table S2. Amino acids and peptides affecting the growth of lrp mutant. Compounds affecting the growth retardation are listed in (1). Compounds affecting the growth enhancement are listed in (2). The growth curves are shown in Figure 2.

Supplemental Table S3. The concentration of metabolites in wild-type strain and the *lrp* mutant. The intracellular levels of metabolites in *E. coli* wild-type BW25113 and its *lrp* mutant JW0872 were determined by TOF/MS analysis. Details of these determinations are described in this table. The results of major metabolites are shown in Figures 3 and 4.

Supplemental Table S1

Lrp-binding sites on the *E. coli* genome (SELEX screening)

Type-A spacers

Position	Function	Operon	Gene	D	Lrp	D	Gene	Operon	Function
42168	predicted transporter	caiTABCDE	caiT	<		>	fixA	fixABCX	electron transfer flavoprotein
83830	leu operon leader peptide	leuLABCD	leuL	<		>	leuO	leuO	transcription factor
255832	aminoacyl-histidine dipeptidase	pepD	pepD	<		>	gpt	gpt	guanine-xanthine phosphoribosyltransferase
310970	transcription factor	matA	matA	<		>	ykgL	ykgL	predicted protein
400468	D-alanine-D-alanine ligase A	ddlA	ddlA	<		>	iraP	iraP	predicted protein
584962	DLP12 prophage OM protease VII	ompT	ompT	<		>	pauD	pauD	Xaa tRNA
632754	predicted oxidoreductase	ydbH-ynbE-ydbL	ybdH	<		>	ybdL	ybdL	methionine aminotransferase
651072	citrate lyase synthetase	citCDEFXG	citC	<		>	dpiB	dpiBA	CitAB TCS sensor kinase
655436	anaerobic C4-dicarboxylate transport	dcuC	dcuC	<		>	pagP	pagP	palmitoyl transferase for Lipid A
675858	conserved protein	ybeQ	ybeQ	<		>	ybeR	ybeR-djlB	predicted protein
784656	conserved protein	ybgS	ybgS	<		>	aroG	aroG	2-dehydro-3-deoxyphosphoheptonate aldolase
815960	NAD(P)-binding Rossman-fold	ybhK	ybhK	<		>	moaA	moaABCDE	molybdopterin biosynthesis protein A
823832	predicted DNase	ybhPON	ybhP	<		>	ybhQ	ybhQ	predicted IM protein
865772	molybdopterin biosynthesis protein	moeAB	moeA	<		>	iaaA	iaaA-gsiABCD	L-asparaginase
915432	aquaporin	aqpZ	aqpZ	<		>	ybjD	ybjD	nucleoside triphosphate hydrolase
931532	thioredoxin reductase	trxB	trxB	<		>	lrp	lrp	transcription factor
1E+06	predicted OM protein	pgaABCD	pgaA	<		>	ycdT	ycdT	predicted diguanylate cyclase
1E+06	e14 prophage IM protein	ymfED	ymfE	<		>	lit	lit	e14 prophage cell death peptidase
1E+06	conserved protein	ycgB	ycgB	<		>	dadA	dadAX	D-amino acid dehydrogenase
1E+06	calcium/sodium:proton antiporter	chaA	chaA	<		>	chaB	chaBC	predicted cation regulator
1E+06	aldehyde-alcohol dehydrogenase	adhE	adhE	<		>	ychE	ychE	predicted IM protein
1E+06	predicted protein	yciN	yciN	<		>	topA	topA	DNA topoisomerase I
1E+06	GGDEF diguanylate cyclase	ydaM	ydaM	<		>	ydaN	ydaN	predicted Zn(II) transporter
2E+06	biofilm-dependent modulation protein	bdm-sra	bdm	<		>	osmC	osmC	osmotically inducible membrane
2E+06	pyridine nucleotide transhydrogenase	pntAB	pntA	<		>	ydgH	ydgH	predicted protein
2E+06	transcription factor	slyA	slyA	<		>	ydhI	ydhIJK	predicted IM protein
2E+06	conserved protein	grxD	grxD	<		>	ydhO	ydhO	predicted lipoprotein
2E+06	conserved protein	ydhQ	ydhQ	<		>	valV	valVW	Val tRNA
2E+06	phosphoenolpyruvate synthase	ppsA	ppsA	<		>	ppsR	ppsR	PEP synthetase regulatory protein
2E+06	succinylornithine transaminase	astCADBE	astC	<		>	xthA	xthA	exonuclease III
2E+06	KpLE2 IS1 repressor InsA	insA	insA	<		>	uspC	uspC	universal stress protein
2E+06	L-arabinose transporter	araFGH	araF	<		>	ftnB	ftnB	predicted ferritin-like protein
2E+06	predicted protein	yecH	yecH	<		>	tyrP	tyrP	tyrosine transporter
2E+06	YedVW TCS response regulator	yedWV	yedW	<		>	yedX	yedX	conserved protein
2E+06	IS5 transposase and trans-activator	insH	insH	<		>	yoeA	yoeA	CP4-44 hemin or colicin receptor
2E+06	predicted protein	yegRZ	yegR	<		>	yegS	yegS	phosphatidylglycerol kinase
2E+06	beta-D-glucoside glucohydrolase	bglX	bglX	<		>	dld	dld	D-lactate dehydrogenase
2E+06	ferredoxin-type protein	napFDAGHBC-ccmABCDEF	napF	<		>	yojO	yojO	hypothetical protein
2E+06	conserved protein	ais	ais	<		>	arnB	arnBCADTEF	UDP-Ara4O aminotransferase
2E+06	transcription factor	lrhA	lrhA	<		>	alaA	alaA	glutamate-pyruvate aminotransferase
2E+06	conserved protein	yfcZ	yfcZ	<		>	fadL	fadL	long-chain fatty acid OM transporter
3E+06	purine nucleoside phosphorylase II	xapAB	xapA	<		>	yfeN	yfeN	conserved OM protein
3E+06	predicted oxidoreductase	aegA	aegA	<		>	narQ	narQ	NarQP(L) TCS sensor kianse
3E+06	Ile tRNA	ileY	ileY	<		>	ygaQ	ygaQ	predicted protein
3E+06	nucleoid-associatd transcription factor	stpA	stpA	<		>	ygaW	ygaW	predicted IM protein
3E+06	HD nuclease-DEAD box helicase	cas3	cas3	<		>	sokX	sokX	sok-related sRNA
3E+06	N-acetylmuramoyl-L-alanine amidase	amiC	amiC	<		>	argA	argA	N-acetylglutamate synthase
3E+06	predicted acyltransferase	ygeF	ygeF	<		>	ygeG	ygeG	predicted transporter
3E+06	predicted protein	ygeK	ygeK	<		>	ygeG	ygeG	predicted chaperone
3E+06	predicted IM protein	yggD	yggD	<		>	metK	metK	methionine adenosyltransferase 1
3E+06	conserved protein	ygiW	ygiW	<		>	qseB	qseBC	QseBC TCS response regulator
3E+06	methyl-accepting aerotaxis sensor	aer	aer	<		>	patA	patA	putrescine aminotransferase
3E+06	transcription factor	tdcABCDEFG	tdcA	<		>	tdcR	tdcR	transcription factor
3E+06	predicted Fe-S oxidoreductase	yhcC	yhcC	<		>	gltB	gltBDF	glutamate synthase
3E+06	transcription factor	nanR	nanR	<		>	dcuD	dcuD	predicted transporter
3E+06	transcription factor	envR	envR	<		>	acrE	acrEF	cytoplasmic membrane lipoprotein
3E+06	peptidyl-prolyl cis-trans isomerase	fkpA	fkpA	<		>	slyX	slyX	conserved protein
4E+06	predicted inner membrane protein	yhgE	yhgE	<		>	pck	pck	phosphoenolpyruvate carboxykinase
4E+06	leucine transporter	livKHMGF	livK	<		>	yhhK	yhhK	conserved protein
4E+06	predicted protein	yhiL	yhiL	<		>	yhiM	yhiM	conserved IM protein
4E+06	IS5 transposase and trans-activator	insH	insH	<		>	slp	slp-dctR	OM lipoprotein
4E+06	gedVW TCS response regulator	gadW	gadW	<		>	gadY	gadY	ncRNA
4E+06	conserved protein	yhjR	yhjR	<		>	bcsE	bcsEFG	conserved protein
4E+06	D-xylose isomerase	xylAB	xylA	<		>	xylF	xylFGHR	D-xylose transporter
4E+06	conserved protein	bax	bax	<		>	malS	malS	alpha-amylase
4E+06	glutamate transporter	gltS	gltS	<		>	xanP	xanP	xanthine NCS2 transporter
4E+06	predicted transcription factor	yieP-hsrA	yieP	<		>	rrsC	rrsC-gltU-rrlC-rrfC	16S rRNA
4E+06	predicted endonuclease	yihG	yihG	<		>	polA	polA	DNA polymerase I
4E+06	GTP-binding protein	yihA	yihA	<		>	csrC	csrC	ncRNA
4E+06	pyridine nucleotide transhydrogenase	sthA	sthA	<		>	fabR	fabR-yijD	transcription factor
4E+06	hypothetical protein	yjbS	yjbS	<		>	aphA	aphA	acid phosphatase/phosphotransferase
4E+06	aspartate ammonia-lyase	aspA-dcuA	aspA	<		>	fxsA	fxsA	IM protein
4E+06	anaerobic fumarate reductase	frdABCD	frdA	<		>	poxA	poxA	predicted lysyl-tRNA synthetase
4E+06	predicted transcriptional factor	ytfJ	ytfJ	<		>	ytfK	ytfK	conserved protein
4E+06	methionine sulfoxide reductase A	msrA	msrA	<		>	ytfM	ytfMNP	OM protein surface antigen
5E+06	KpLE2 IS4 transposase	insG	insG	<		>	yjhB	yjhBC	KpLE2 phage transporter
5E+06	predicted protein	yjiC	yjiC	<		>	iraD	iraD	RpoS proteolysis inhibitor
5E+06	nucleoid-associated transcription factor	rob	rob	<		>	creA	creABCD	conserved protein

Total, 78 peaks (target operons, 156)

Type-B spacers

Position	Function	Operon	Gene	D	Lrp	D	Gene	Operon	Function
9262	transaldolase B		talB	>		>	mog	mog	predicted molybdochelatase

85536	transcriptional factor		leuO	>		>	ilvI	ilvIH	acetolactate synthase III
152844	predicted fimbrial-like adhesin protein	yadMLKC	yadM	<		<	htrE		predicted OM usher protein
155442	predicted OM usher protein	htrE	htrE	<		<	ecpD		predicted periplasmic pilin chaperone
236848	DNA polymerase III		dnaQ	>		>	aspV		Asp tRNA
251970	DNA polymerase IV		dinB	>		>	yafN	yafNOP	YafO-YafN toxin-antitoxin
317836	predicted protein	ykgIB	ykgI	<		<	ykgC		predicted oxidoreductase
320346	transcription factor		ykgD	>		>	ykgE	ykgEFG	predicted oxidoreductase
389160	porphobilinogen synthase	hemB	hemB	<		<	insF		IS3 element protein InsF
418534	PhoBR TCS sensor kinase		phoR	>		>	brnQ	brnQ-proY	branched amino acid transporter
433872	diaminohydroxyphosphoribosylaminopyrimidine deaminase-amino-(5-phosphoribosylamino)uracil reductase		ribD	>		>	ribE	ribE-nusB-thiL-pgpA	riboflavin synthase beta chain
467530	thiamin pyrimidine pyrophosphate hydrolase		cof	>		>	ybaO	ybaO	predicted transcription factor
479234	maltose O-acetyltransferase	maa	maa	<		<	hha		H-NS regulator
530448	tRNA 2-selenouridine synthase	ybbB	ybbB	<		<	allS		transcription factor
536860	tartronate semialdehyde reductase		glxR	>		>	ybbW	ybbW-allB-ybbY-glxK	predicted allantoin transporter
567532	IS3 element protein InsF		insF	>		>	emrE	emrE	DLP12 multidrug resistance protein
569664	DLP12 prophage recombinase		ybcK	>		>	ybcL	ybcLM	DLP12 kinase inhibitor
603938	mechanosensitive channel	mscM	mscM	<		<	nfsB		dihydropteridine reductase
659532	lipoate synthase	lipA	lipA	<		<	ybeF		predicted transcription factor
696540	Met tRNA	metT-leuW-glnUW-metU-glnVX	metT	<		<	asnB		asparagine synthetase B
735642	predicted protein		ybfC	>		>	ybfQ	ybfQ	predicted transposase
736068	predicted transposase		ybfQ	>		>	ybfL	ybfL	predicted transposase
802538	predicted transporter		ybhI	>		>	ybhJ	ybhJ	predicted hydratase
837438	predicted protein	ybiJ	ybiJ	<		<	ybiI		conserved protein
892742	predicted oxidoreductase		ybjN	>		>	potF	potFGHI	putrescine transporter
903170	arginine transporter	artPIQM	artP	<		<	ybjP		predicted lipoprotein
936430	chromosome resolution and		ftsK	>		>	lolA	lolA-rarA	chaperone for lipoproteins
938542	recombination protein		rarA	>		>	serS	serS	seryl-tRNA synthetase
946370	predicted transporter		ycaD	>		>	ycaM	ycaM	predicted transporter
956734	conserved inner membrane protein		ycaP	>		>	serC	serC-aroA	phosphohydroxythreonine aminotransferase
985134	aspartate aminotransferase,	aspC	aspC	<		<	ompF		OM porin 1a
986550	OM porin 1a	ompF	ompF	<		<	asnS		asparaginyl tRNA synthetase
1E+06	DNA-binding protein	hspQ	hspQ	<		<	yccW		predicted methyltransferase
1E+06	conserved protein		efeB	>		>	phoH	phoH	nucleoside triphosphate hydrolase
1E+06	predicted protein	bssS	bssS	<		<	dinI		DNA damage-inducible protein I
1E+06	predicted lipoprotein	yceB	yceB	<		<	grxB		glutaredoxin 2
1E+06	e14 prophage methyltransferase	ymfD	ymfD	<		<	ymfE		e14 prophage IM protein
1E+06	RpoS proteolysis inhibitor	iraM	iraM	<		<	ycgX		predicted protein
1E+06	predicted transcription factor	ycgE	ycgE	<		<	ycgF		FAD-binding phosphodiesterase
1E+06	conserved IM protein		ycgG	>		>	ymgF	ymgF	predicted protein
1E+06	predicted adhesin	ycgV	ycgV	<		<	ychF		predicted GTP-binding protein
1E+06	predicted transcription factor		ychA	>		>	kdsA	kdsA	D-manno-octulosonate 8-phosphate synthase
1E+06	nitrate/nitrite transporter		narK	>		>	narG	narGHJI	nitrate reductase 1
1E+06	predicted IM protein		ychE	>		>	oppA	oppABCDF	oligopeptide transporter
1E+06	predicted IM protein		yciQ	>		>	rluB	rluB	23S rRNA pseudouridylate synthase
1E+06	DNA topoisomerase It		topA	>		>	cysB	cysB	transcription factor
1E+06	transcription factor		cysB	>		>	ymiA	ymiA-yciX	hypothetical protein
1E+06	hypothetical protein	yciZT	yciZ	<		<	gmr		RNase II modlator
1E+06	RNase II stability modulator	gmr	gmr	<		<	rnb		ribonuclease II
1E+06	conserved IM protein		ycjF	>		>	tyrR	tyrR	transcription factor
1E+06	Rac prophage site-specific	pinR	pinR	<		<	ynaE		Rac transcription factor
2E+06	SAM-dependent methyltransferase		tehB	>		>	ydcL	ydcL	predicted lipoprotein
2E+06	nitrate/nitrite transporter	narU	narU	<		<	yddJ		predicted protein
2E+06	predicted protein	yddJ	yddJ	<		<	yddG		methyl viologen efflux pump
2E+06	diguanylate cyclase	dosCP	dosC	<		<	yddW		predicted liprotein
2E+06	glutamate decarboxylase B	gadBC	gadB	<		<	pqqL		predicted peptidase
2E+06	conserved protein	ydeN	ydeN	<		<	ydeO		transcription factor
2E+06	predicted lipoprotein	ydeK	ydeK	<		<	lsrK		autoinducer-2 (AI-2) kinase
2E+06	predicted diguanylate cyclase	yneF	yneF	<		<	yneG		conserved protein
2E+06	conserved protein	ydeH	ydeH	<		<	ydeI		conserved protein
2E+06	Qin prophage transcription fctor		ydfK	>		>	pinQ	pinQ	Qin site-specific recombinase
2E+06	predicted protein		ydgH	>		>	ydgI	ydgI-folM	arginine/ornithine antiporter transporter
2E+06	DNA glycosylase-endonuclease III		nth	>		>	tppB	tppB	predicted transporter
2E+06	conserved protein	ydiV	ydiV	<		<	nlpC		predicted lipoprotein
2E+06	predicted diguanylate cyclase		yeaI	>		>	yeaJ	yeaJ	predicted diguanylate cyclase
2E+06	neutral amino-acid efflux system	leuE	leuE	<		<	yeaT		transcription factor
2E+06	predicted NUDIX hydrolase		nudL	>		>	sdaA	sdaA	L-serine deaminase I
2E+06	ncRNA		rseX	>		>	hchA	hchA	Hsp31 molecular chaperone
2E+06	AMP nucleosidase		amn	>		>	yeeN	yeeN	conserved protein
2E+06	cobinamide kinase-	cobUST	cobU	<		<	insH		IS5 transposase and trans-activator
2E+06	predicted IM protein	yeeED	yeeE	<		<	yeeF		predicted amino-acid transporter
2E+06	predicted peptidase		yegQ	>		>	cyaR	cyaR	ncRNA
2E+06	galactitol-specific PTS IIA	gatABCD	gatA	<		<	gatZ		D-tagatose 1,6-bisphosphate aldolase
2E+06	predicted protein		yehK	>		>	yehL	yehLMPQ	predicted transporter subunit
2E+06	conserved protein	yehS	yehS	<		<	yehT		YehUT TCS response regulator
2E+06	predicted IM protein		yeiS	>		>	yeiT	yeiTA	Dihydropyrimidine dehydrogenase
2E+06	endonuclease IV		nfo	>		>	yeiI	yeiI	predicted kinase
2E+06	undecaprenyl pyrophosphate		lpxT	>		>	spr	spr	predicted peptidase
2E+06	hypothetical protein		yojO	>		>	eco	eco	serine protease inhibitor ecptin
2E+06	ncRNA		micF	>		>	rcsD	rcsDB	RcsBC TCS phospho transfer
2E+06	predicted protein	yfaQP	yfaQ	<		<	yfaT		predicted protein
2E+06	NADH:ubiquinone oxidoreductase,	nuoABCEFG HIJKLMN	nuoA	<		<	lrhA		transcription factor
2E+06	phosphate acetyltransferase		pta	>		>	yfcC	yfcC	predicted IM protein
3E+06	predicted peptidase		yfhR	>		>	csiE	csiE	stationary phase inducible protein
3E+06	16S rRNA	rrsG-gltW-rrlG-rrfG	rrsG	<		<	clpB		protein disaggregation chaperone
3E+06	cold shock protein		raiA	>		>	pheL	pheLA	pheA gene leader peptide

3E+06	ribonucleoside-diphosphate reductase		***nrdF***	>		>	***proV***	*proVWX*	glycine betaine transporter
3E+06	cascade subunit A	*casABCDE12*	***casA***	<		<	***cas3***		HD nuclease-DEAD box helicase
3E+06	predicted D-glucarate transporter	*gudPXD*	***gudP***	<		<	***ygcA***		predicted flavoprotein
3E+06	conserved protein		***ygdH***	>		>	***sdaC***	*sdaCB*	predicted serine transporter
3E+06	predicted chaperone		***ygeG***	>		>	***ygeH***	*ygeH*	transcription factor
3E+06	predicted transporter		***ygfO***	>		>	***guaD***	*guaD-ygfQ*	guanine deaminase
3E+06	aminomethyltransferase,	*gcvTHP*	***gcvT***	<		<	***visC***		predicted oxidoreductase
3E+06	D-3-phosphoglycerate dehydrogenase	*serA*	***serA***	<		<	***rpiA***		ribose 5-phosphate isomerase
3E+06	periplasmic L-asparaginase II	*ansB*	***ansB***	<		<	***yggN***		predicted protein
3E+06	predicted IM lipoprotein	*yghJ*	***yghJ***	<		<	***glcA***		glycolate transporter
3E+06	glycolate transporter	*glcA*	***glcA***	<		<	***glcB***		malate synthase G
3E+06	phosphate transporter	*pitB*	***pitB***	<		<	***gsp***		glutathionylspermidine synthetase
3E+06	conserved protein		***ygiC***	>		>	***ygiL***	*ygiL*	predicted fimbrial-like adhesin
3E+06	transcription factor		***tdcR***	>		>	***yhaB***	*yhaBC*	predicted protein
3E+06	glutamate synthase, 4Fe-4S protein		***gltD***	>		>	***gltF***	*gltF*	periplasmic protein
3E+06	transcription factor		***argR***	>		>	***yhcN***	*yhcN*	conserved protein
3E+06	predicted OM protein		***yhdV***	>		>	***yhdW***	*yhdWXYZ*	YhdW
3E+06	conserved protein	*yhdN-zntR*	***yhdN***	<		<	***rplQ***		50S ribosomal subunit protein L17
3E+06	50S ribosome protein L6	*rplFR-rpsE-rpmD-rplO-secY-rpmJ*	***rplF***	<		<	***rpsH***		30S ribosomal subunit protein S8
4E+06	conserved protein		***yhhZ***	>		>	***yrhA***	*yrhA-insA-6AB-6B-6*	conserved protein
4E+06	leucine/isoleucine/valine transporter	*livJ*	***livJ***	<		<	***rpoH***		RNA polymerase RpoH sigma
4E+06	predicted protein		***yhhH***	>		>	***yhhI***	*yhhI*	predicted transposase
4E+06	universal stress protein		***uspA***	>		>	***dtpB***	*dtpB*	predicted transporter
4E+06	arsenate reductase		***arsC***	>		>	***yhiS***	*yhiS*	predicted protein
4E+06	conserved IM protein		***yhjD***	>		>	***yhjE***	*yhjE*	predicted transporter
4E+06	predicted OM biogenesis protein	*yhjG*	***yhjG***	<		<	***yhjH***		flagellar function EAL regulator
4E+06	dipeptide transporter	*dppABCDF*	***dppA***	<		<	***proK***		Pro tRNA
4E+06	alpha-amylase		***malS***	>		>	***avtA***	*avtA*	valine-pyruvate aminotransferase
4E+06	conserved IM protein	*yiaWV*	***yiaW***	<		<	***aldB***		aldehyde dehydrogenase B
4E+06	Fe-containing alcohol dehydrogenase	*yiaY*	***yiaY***	<		<	***selB***		selecnoCys-tRNA translation factor
4E+06	glycine C-acetyltransferase	*kbl-tdh*	***kbl***	<		<	***htrL***		predicted protein
4E+06	LPS heptosyl transferase I		***rfaC***	>		>	***rfaL***	*rfaL*	O-antigen ligase
4E+06	lexA-regulated toxic peptide		***tisB***	>		>	***emrD***	*emrD*	multidrug efflux system protein
4E+06	GTPase		***mnmE***	>		>	***tnaC***	*tnaCAB*	tryptophanase leader peptide
4E+06	tryptophanase leader peptide		***tnaC***	>		>	***tnaA***	*tnaAB*	tryptophanase/cysteine desulfhydrase
4E+06	glucosamine-1-phosphate acetyl transferase	*glmUS*	***glmU***	<		<	***atpC***		F1 membrane-bound ATP synthase
4E+06	disulfide isomerase I		***dsbA***	>		>	***yihF***	*yihF*	conserved protein
4E+06	predicted acetyltransferase		***yiiD***	>		>	***yiiE***	*yiiE*	predicted transcriptional regulator
4E+06	homoserine O-transsuccinylase		***metA***	>		>	***aceB***	*aceBAK*	malate synthase A
4E+06	ankyrin repeat protein	*arpA*	***arpA***	<		<	***iclR***		DNA-binding transcriptional repressor
4E+06	SOS response protein		***dinF***	>		>	***yjbJ***	*yjbJ*	predicted stress response protein
4E+06	heme lyase		***nrfG***	>		>	***gltP***	*gltP*	glutamate/aspartate:proton symporter
4E+06	predicted alkyl sulfatase	*yjcS*	***yjcS***	<		<	***alsK***		D-allose kinase
4E+06	transcription factor	*adiY*	***adiY***	<		<	***adiA***		biodegradative arginine decarboxylase
4E+06	C4-dicarboxylate antiporter	*dcuB-fumB*	***dcuB***	<		<	***dcuR***		DcuSR TCS responsee regulator
4E+06	lysine tRNA synthetase, inducible	*lysU*	***lysU***	<		<	***yjdL***		predicted transporter
4E+06	predicted lysine/cadaverine transporter	*cadBA*	***cadB***	<		<	***cadC***		DNA-binding transcriptional activator
4E+06	predicted lysyl-tRNA synthetase		***poxA***	>		>	***yjeM***	*yjeM*	predicted transporter
4E+06	conserved protein		***yjfM***	>		>	***yjfC***	*yjfC*	predicted synthetase/amidase
4E+06	peptidyl-prolyl cis-trans isomerase		***fklB***	>		>	***cycA***	*cycA*	D-alanine/D-serine/glycine transporter
4E+06	KpLE2 IS2 insertion transposase'		***insD***	>		>	***yjgW***	*yjgW*	KpLE2 phage-like element
5E+06	KpLE2 transcription factor	*yjhIHG*	***yjhI***	<		<	***sgcR***		KpLE2 transcription factor
5E+06	*fimA* switch tyrosine recombinase		***fimE***	>		>	***fimA***	*fimAICDFGH*	major type 1 subunit fimbrin (pilin)
5E+06	peptide chain release factor RF-3		***prfC***	>		>	***osmY***	*osmY*	periplasmic protein

Total 140 peaks (target operons, 140)

Type-C spacers

Position	Function	Operon	*Gene*	D	Lrp	D	Gene	Operon	Function
238230	predicted aminopeptidase		***yafT***	>		<	***ykfM***		hypothetical protein
583650	DLP12 transcriptional activator		***appY***	>		<	***ompT***		DLP12 OM protease VII
1E+06	cation transport regulator		***chaC***	>		<	***ychN***		conserved protein
1E+06	IM phosphatase		***ynbD***	>		<	***azoR***		NADH-azoreductase
3E+06	RNA-binding protein		***yhbY***	>		<	***greA***		transcription elongation factor
4E+06	sn-glycerol-3-phosphate		***glpD***	>		<	***glgP***		glycogen phosphorylase
4E+06	IM protein		***yhiM***	>		<	***yhiN***		oxidoreductase
4E+06	Trp tRNA		***trpT***	>		<	***hdfR***		transcription factor
4E+06	heat shock protein		***yjaZ***	>		<	***sroH***		SroH small RNA
5E+06	ncRNA		***symR***	>		<	***hsdS***		HsdM and HsdR regulator

Total, 10 peaks

Inside ORF

Position	Function	Operon	*Gene*	D	Lrp	D	Gene	Operon	Function
168	predicted protein		***yjjY***	>	***yjtD***	>	***thrL***	***thrLABC***	*thr* operon leader peptide
102758	UDP-N-acetylmuramate:alanine ligase		***murC***	>	***ddlB***	>	***ftsQ***	***ftsQAZ-lpxC***	cell division septum protein
223368	DL-methionine transporter	***metNIQ***	***metN***	<	***gmhB***	>	***rrsH***	***rrsH-ileV-alaV-rrlH-rrfH***	16S rRNA
296170	CP4-6 prophage protein	***yagN***	***yagN***	<	***intF***	<	***yagP***		predicted transcriptional regulator
302552	predicted xanthine dehydrogenase	***yagTSRQ***	***yagT***	<	***yagU***	<	***ykgJ***		predicted ferredoxin
360530	predicted cyanate transporter		***cynX***	>	***lacA***	<	***lacY***		lactose/galactose transporter
515656	membrane-associated protease	***qmcA-ybbJ***	***qmcA***	<	***ybbL***	>	***ybbM***	***ybbM***	predicted IM protein
526956	rhsD element protein		***rhsD***	>	***ybbC***	>	***ylbH***	***ylbH-ybbD***	conserved protein
568958	DLP12 MD resistance protein		***emrE***	>	***ybcK***	>	***ybcL***	***ybcLM***	DLP12 prophage kinase inhibitor
578634	DLP12 prophage lipoprotein	***borD***	***borD***	<	***ybcV***	>	***ybcW***	***ybcW***	DLP12 prophage protein
636446	conserved protein	***ybdN***	***ybdN***	<	***ybdO***	<	***dsbG***		disulfide isomerase
677132	predicted protein		***ybeR***	>	***djlB***	<	***ybeT***		conserved OM protein
737356	predicted transposase		***ybfQ***	>	***ybfD***	>	***ybgA***	***ybgA-phr***	conserved protein
776330	TolAQR complex protein		***tolR***	>	***tolA***	>	***tolB***	***tolB-pal-ybgF***	periplasmic protein
811762	biotin synthesis methltransferase		***bioC***	>	***bioD***	<	***ybhU***		hypothetical protein
906136	NAD(P)H-binding oxidoreductase	***ybjS***	***ybjS***	<	***ybjT***	<	***ltaE***		L-allo-threonine aldolase
909154	L-allo-threonine aldolase	***ltaE-ybjT***	***ltaE***	<	***poxB***	<	***hcr***		HCP oxidoreductase
921444	macrolide transporter		***macA***	>	***macB***	<	***cspD***		cold shock protein homolog
988572	asparaginyl tRNA synthetase	***asnS***	***asnS***	<	***pncB***	>	***pepN***	***pepN***	aminopeptidase N

1E+06	predicted hydrolase		ymdC	>	mdo	>	mdoG	mdoGH	glucan biosynthesis protein
1E+06	RpoS proteolysis inhibitor	iraM	iraM	<	ycgX	<	ycgE		predicted transcription factor
1E+06	Tyr tRNA	tyrTV-tpr	tyrT	<	purU	<	ychJ		conserved protein
1E+06	aconitate hydratase 1		acnA	>	ribA	>	pgpB	pgpB	phosphatidylglycerophosphatase B
1E+06	conserved IM protein		ycjF	>	tyrR	<	tpx		lipid hydroperoxide peroxidase
1E+06	fermentative D-lactate dehydrogenase	ldhA	ldhA	<	ydbH	>	ynbE	ynbE-ydbL	predicted lipoprotein
1E+06	L7/L12-serine acetyltransferase		rimL	>	ydcK	>	tehA	tehAB	proflavin transporter
2E+06	predicted protein		ydcD	>	ydcC	>	pptA	pptA	4-oxalocrotonate tautomerase
2E+06	predicted peptidase	pqqL	pqqL	<	yddB	<	yddA		ABC-family MD transporter
2E+06	autoinducer-2 (AI-2) kinase	lsrK	lsrK	<	lsrR	>	lsrA	lsrACDBFG-	ABC-family AI2 transporter
2E+06	cysteine acetyl-serine efflux system	eamA	eamA	<	ydeE	>	yneM	yneM	hypothetical protein
2E+06	predicted protein		ynfD	>	ynfE	>	ynfF	ynfFGH-dmsD	oxidoreductase subunit
2E+06	OM porin protein	uidBC	uidB	<	uidA	<	uidR		transcription factor
2E+06	threonyl-tRNA synthetase	thrS-infC-rpmI-rplT-pheMST-ihfA	thrS	<	arpB	>	yniD	yniD	predicted protein
2E+06	predicted acyltransferase		yedN	<	yedN 1	<	yedM		conserved protein
2E+06	IS5 transposase and trans-activator	insH	insH	<	wbbK	<	wbbJ		predicted acyl transferase
2E+06	conserved protein	wbbIJK	wbbI	<	rfc	<	glf		UDP-galactopyranose mutase
2E+06	O-antigen polymerase	rfc	rfc	<	glf	<	rfbX		polisoprenol-linked O-antigen
2E+06	UDP-galactopyranose mutase	glf-rfc	glf	<	rfbX	<	rfbC		dTDP-4-deoxyrhamnose-3,5-
2E+06	predicted subunit with GalU	galF	galF	<	wcaM	<	wcaL		predicted glycosyl transferase
2E+06	IS3 element protein InsF		insF	>	gatR	<	gatD		galactitol-1-phosphate dehydrogenase
2E+06	predicted OM protein	yehBA	yehB	<	yehC	<	yehD		predicted fimbrial-like adhesin protein
2E+06	predicted protein	yfaTS	yfaT	<	yfaA	<	gyrA		DNA gyrase (type II topoisomerase)
2E+06	predicted NUDIX hydrolase		nudI	>	ais	>	arnB	arnBCADTEF	Ur (pentapyranosyl diphosphate) aminotransferase
2E+06	enoyl-CoA hydratase and epimerasee	fadJ	fadJ	<	fadI	<	yfcZ		conserved protein
2E+06	predicted CoA-transferase	yfdE	yfdE	<	yfdV	<	oxc		predicted oxalyl-CoA decarboxylase
3E+06	transaldolase A		talA	>	tktB	<	ypfG		predicted protein
3E+06	predicted protein		yfiR	>	yfiN	>	yfiB	yfiB	predicted OM lipoprotein
3E+06	CP4-57 prophage protein	ypjM	ypjM	<	yfjW	>	yfjX	yfjXY-ypjJ-yfjZ-ypjF	CP4-57 antirestriction protein
3E+06	alanyl-tRNA synthetase	alaS	alaS	<	recX	<	recA		recombination protein
3E+06	enolase	eno	eno	<	pyrG	<	mazG		NTP pyrophosphohydrolase
3E+06	L-fuculose-1-phosphate aldolase	fucAO	fucA	<	fucP	>	fucI	fucIKUR	L-fucose isomerase
3E+06	predicted chaperone		ygeG	>	ygeH	>	ygeI	ygeI	predicted protein
3E+06	conserved protein	ygiQ	ygiQ	<	ftsP	<	plsC		acyl-glycerol-3-phosphate
3E+06	conserved IM protein		ygiH	>	ttdR	>	ttdA	ttdABT	L-tartrate dehydratase
3E+06	conserved IM protein		ygjA	>	ygjB	>	ygjC	ygjCDEK	conserved protein
3E+06	predicted protein		yhaB	>	yhaC	<	rnpB		ncRNA
4E+06	heat shock protein Hsp33		hslO	>	yhgE	>	pck	pck	phosphoenolpyruvate carboxykinase
4E+06	predicted acetyltransferase		yhhY	>	yhhZ	>	yrhA	yrhA-insA-6AB	conserved protein
4E+06	*rhsB* element core protein RshB		rhsB	>	yhhH	>	yhhI	yhhI	predicted transposase
4E+06	predicted transposase		yhhI	>	yhhJ	<	rbbA		ribosome-associated ATPase
4E+06	predicted HlyD family secretion protein	yhiI-rbbA-	yhiI	<	yhiJ	<	yhiK		predicted protein
4E+06	MgtC-family IM transporterr	yhiD	yhiD	<	hdeB	<	hdeA		acid-resistance protein
4E+06	multidrug transporter		mdtF	>	gadW	>	gadY	gadY	ncRNA
4E+06	ncRNA		rdlD	>	yhjV	<	dppF		dipeptide transporter
4E+06	predicted transporter	yhjX	yhjX	<	yhjY	>	tag	tag-yiaC	3-methyl-adenine DNA glycosylase I
4E+06	predicted protein	ysaB	ysaB	<	wecH	<	yiaA		conserved IM protein
4E+06	predicted glutathione S-transferase	yibF	yibF	<	rhsA	>	yibA	yibA	lyase containing HEAT-repeat
4E+06	*rhsA* element core protein		rhsA	>	yibA	>	yibG	yibG	conserved protein
4E+06	LPS core biosynthesis	waaK	waaK	<	rfaZ	<	rfaY		LP core biosynthesis protein
4E+06	UDP-D-galactose galactosyltransferase	waaBIJY-rfaZ-waaK	waaB	<	rfaS	<	rfaP		LP kinase
4E+06	predicted alpha-glucosidase	yicI	yicI	<	yicJ	>	selC	selC	Sec tRNA
4E+06	conserved IM protein	yigF	yigF	<	yigG	<	rarD		chloramphenical resistance permease
4E+06	OM porin protein	ompL	ompL	<	yihO	<	yihP		predicted transporter
4E+06	isocitrate dehydrogenase kinase		aceK	>	arpA	<	iclR		transcription factor
4E+06	predicted cation/proton antiporter		yjcE	>	yjcF	<	actP		acetate transporter
4E+06	formate-dependent nitrite reductase		nrfD	>	nrfE	>	nrfF	nrfFG	heme lyase (NrfEFG)
4E+06	transcription factor	melR	melR	<	melA	>	melB	melB	melibiose:sodium symporter
4E+06	predicted protein		yjdO	>	lysU	<	yjdL		predicted transporter
4E+06	predicted lysyl-tRNA synthetase		poxA	>	yjeM	>	yjeN	yjeNO	predicted protein
4E+06	cell envelope opacity protein	ytfB	ytfB	<	fklB	>	cycA	cycA	D-alanine/D-serine/glycine transporter
4E+06	predicted IM protein	ytfF	ytfF	<	ytfG	>	ytfH	ytfH	transcription factor
4E+06	ABC-family sugar transporter y		yjfF	>	fbp	>	mpl	mpl	UDP-N-acetylmuramate ligase
5E+06	KpLE2 transposase	insG	insG	<	yjhB	>	yjhC	yjhC	KpLE2 phage oxidoreductase
5E+06	KpLE2 phage transporter		yjhB	>	yjhC	>	ythA	ythA	small predicted membrane protein
5E+06	ncRNA		symR	>	hsdS	<	hsdM		DNA methylase M
5E+06	lipoate-protein ligase A	lplA	lplA	<	ytjB	>	serB	serB-radA-	3-phosphoserine phosphatase

Total, 86 (possible targets, 89)

Supplemental Table S2

Amino acids and peptides affecting the growth of *lrp* mutant

1) Compounds affecting the growth retardation

Amino acids	L-Ala, L-Cys, L-Gly, L-Ser, L-Trp<br>D-Ala, D-Ser
Peptides	Ala-Gly, Ala-His, Ala-Thr, Gly-Asn, Ala-Ala, Ala-Arg, Ala-Asn, Ala-His, Ala-Ser, Ala-Thr, Arg-Ser, Gln-Gln, Gly-Ala, Gly-Arg, Gly-Gly, Gly-Lys, Gly-Met, Gly-Ser, Gly-Thr, Leu-Ala, Leu-Arg, Leu-Glu, Met-Gly, Phe-Ala, Phe-Gly, Phe-Ser, Ser-Ala, Ser-Gly, Ser-His, Ser-Met, Ser-Phe, Ser-Ser, Thr-Ala, Thr-Gly, Trp-Ala, Trp-Leu, Trp-Ser, Tyr-Ala, Tyr-Gly, Lys-Gly, Met-Thr, Ser-Asn, Ala-Ala-Ala, Gly-Gly-Ile, Gly-Gly-Leu, Gly-Gly-Phe

2) Compounds affecting the growth enhancement

Amino acids	None
Peptides	Pro-Phe, Pro-Pro, Pro-Tyr, Tyr-Glu, His-Glu, Phe-Asp, Phe-Glu, Pro-Glu, Pro-Ile, Pro-Trp

Supplemental Table S3

Concentration of major intermediate metabolites in wild-type and lrp mutant (measured by TOF/MS)

Annotation Name	Ave m/z	Conc (pmol/OD/ml) WT	lrp mutant	ratio (lrp/wt)
Glyoxylate	72.996	0.0	0.0	–
Glycolate	75.011	0.0	0.0	–
Gly	76.042	319.3	833.1	2.61
Pyruvate	87.009	0.0	0.0	–
Lactate	89.026	4234.6	2227.6	0.53
Putrescine(1,4-Butanediamine)	89.109	282.6	96.0	0.34
beta-Ala	90.057	9.2	3.4	0.38
Ala	90.058	192.6	87.1	0.45
Spermine divalent	102.118	0.0	0.0	–
GABA	104.073	55.8	6.3	0.11
2-Aminobutyrate	104.073	51.4	25.7	0.50
Ser	106.051	121.4	70.5	0.58
Hypotaurine	110.027	0.0	0.0	–
Cytosine	112.051	1.2	1.4	1.12
Uracil	113.035	0.0	0.0	–
Carnosine divalent	114.062	0.0	0.0	–
Fumarate	115.005	197.6	219.0	1.11
2-Oxoisopentanoate	115.042	0.0	0.0	–
Pro	116.071	54.7	32.3	0.59
Succinate	117.020	228.5	195.5	0.86
Val	118.087	162.9	162.2	1.00
Thr	120.066	150.1	73.6	0.49
Homoserine	120.066	36.5	23.0	0.63
Cys	122.028	0.0	0.0	–
Taurine	126.022	0.0	0.0	–
Hydroxyproline	132.066	0.0	0.0	–
Creatine	132.077	0.0	0.0	–
Ile	132.102	11.8	7.5	0.63
Leu	132.102	43.9	47.8	1.09
Malate	133.015	479.3	448.1	0.93
Asn	133.061	81.4	74.5	0.91
Ornithine	133.097	5.9	4.4	0.74
Asp	134.044	244.1	86.9	0.36
Homocysteine	136.042	0.0	0.0	–
Adenine	136.063	94.3	49.1	0.52
Hypoxanthine	137.046	204.5	127.5	0.62
Anthranilate	138.055	0.0	0.0	–
Tyramine	138.092	1.6	1.0	0.59
2-Oxoglutarate	145.015	0.0	0.0	–
Spermidine	146.164	1.4	0.0	0.00
2-Hydroxyglutarate	147.031	0.0	25.7	–
Gln	147.077	339.5	82.1	0.24
Lys	147.113	335.8	407.3	1.21
Glu	148.061	3598.3	753.6	0.21
Met	150.059	24.9	23.9	0.96
Cysteine sulfinate	152.004	0.0	0.0	–
Guanine	152.057	29.7	23.5	0.79
His	156.077	30.4	31.3	1.03
Phe	166.086	34.4	52.9	1.54
PEP	166.976	483.3	366.9	0.76
;G3P	168.992	0.0	0.0	–
DHAP	168.992	76.0	16.5	0.22

Glycerophosphate	171.007	337.5	225.6	0.67
cis-Aconitate	173.010	6.2	2.5	0.41
Arg	175.120	143.9	105.3	0.73
Citrulline	176.103	9.1	11.4	1.25
Tyr	182.081	115.9	210.6	1.82
3PG;2PG	184.987	1408.5	1135.0	0.81
2PG	184.987	0.0	0.0	–
Citrate	191.021	38.4	45.5	1.19
Isocitrate	191.022	0.0	0.0	–
S-Adenosylhomocysteine	193.069	2.0	1.7	0.82
Gluconate	195.052	0.0	0.0	–
DOPA	198.076	0.0	0.0	–
S-Adenosylmethionine	200.078	39.9	36.6	0.92
Trp	205.098	1.9	3.7	1.95
Cystathionine	223.074	0.0	0.0	–
R5P	229.013	45.4	30.2	0.67
Ru5P	229.013	76.8	31.6	0.41
gamma-Glu-2-aminobutyrate	233.113	0.0	2.6	–
Cytidine	244.094	0.0	0.8	–
Uridine	245.077	0.0	0.0	–
gamma-Glu-cys	251.071	0.0	0.0	–
G6P	259.023	174.0	108.2	0.62
F6P	259.024	52.7	39.6	0.75
G1P	259.024	76.8	34.9	0.45
2,3-DPG	264.954	34.3	8.5	0.25
Adenosine	268.104	4.9	10.2	2.09
Inosine	269.088	11.0	16.0	1.45
6-Phosphogluconate	275.019	18.6	9.8	0.52
Guanosine	284.100	4.9	6.1	1.23
S7P	289.035	66.1	32.8	0.50
Ophthalmate	290.135	156.5	291.8	1.86
Glutathione(ox)	307.084	1185.5	821.3	0.69
Glutathione(red)	308.092	0.0	0.0	–
dTMP	321.050	70.9	68.2	0.96
CMP	322.046	82.7	35.9	0.43
cAMP	328.046	31.8	31.1	0.98
F1,6P	338.990	129.1	32.5	0.25
cGMP	344.042	0.0	0.0	–
AMP	346.057	175.0	196.2	1.12
IMP	347.041	77.1	202.5	2.63
GMP	362.052	14.5	16.5	1.14
NADPH	371.540	0.0	0.0	–
CoA	382.558	0.0	0.0	–
PRPP	388.947	0.0	0.0	–
dTDP	401.017	54.8	34.5	0.63
CDP	402.012	124.6	54.4	0.44
Acetyl CoA	403.559	20.5	1.0	0.05
Malonyl CoA	425.554	0.0	0.0	–
ADP	426.024	751.1	654.7	0.87
Succinyl CoA	432.563	0.0	0.0	–
GDP	442.018	295.7	211.1	0.71
dCTP	465.984	108.6	66.7	0.61
dTTP	480.984	209.7	94.2	0.45
CTP	481.979	627.1	244.1	0.39
UTP	482.962	1638.6	925.4	0.56
dATP	489.995	209.2	115.2	0.55
ATP	505.990	3894.2	2378.2	0.61

GTP	521.985	1945.9	1020.3	0.52
NAD+	662.103	975.9	580.3	0.59
NADH	664.119	0.0	0.0	−
NADP+	742.070	413.2	218.6	0.53
FAD	784.153	17.7	0.0	0.00
